# Real-world clinical practice and outcomes in treating stage III non-small cell lung cancer: KINDLE-Asia subset

**DOI:** 10.3389/fonc.2023.1117348

**Published:** 2023-03-27

**Authors:** Kumar Prabhash, Daniel Shao Weng Tan, Ross A. Soo, Piyada Sitthideatphaiboon, Yuh Min Chen, Pei Jye Voon, Elisna Syahruddin, Sojung Chu, Reto Huggenberger, Byoung-Chul Cho

**Affiliations:** ^1^ Department of Medical Oncology, Tata Memorial Hospital, Mumbai, Maharashtra, India; ^2^ Division of Medical Oncology, National Cancer Centre, Singapore, Singapore; ^3^ Department of Haematology-Oncology, National University Cancer Institute, Singapore, Singapore; ^4^ Division of Medical Oncology, Department of Medicine, Faculty of Medicine, Chulalongkorn University and King Chulalongkorn Memorial Hospital, Bangkok, Thailand; ^5^ Taipei Veterans General Hospital, School of Medicine, National Yang-Ming Medical University, Taipei City, Taiwan; ^6^ Department of Radiotherapy and Oncology, Hospital Umum Sarawak, Kuching, Sarawak, Malaysia; ^7^ Department of Pulmonology and Respiratory Medicine, Faculty of Medicine, Universitas Indonesia, Persahabatan Hospital, Jakarta, Indonesia; ^8^ Medical Affairs, AstraZeneca, Seoul, Republic of Korea; ^9^ Medical Affairs, AstraZeneca, Baar, Switzerland; ^10^ Division of Medical Oncology, Yonsei Cancer Centre, Yonsei University College of Medicine, Seoul, Republic of Korea

**Keywords:** lung cancer, EGFR mutation, stage III NSCLC, adenocarcinoma, targeted therapy, concurrent chemoradiotherapy (CCRT)

## Abstract

**Introduction:**

Stage III non-small cell lung cancer (NSCLC) is a heterogeneous disease requiring multimodal treatment approaches. KINDLE-Asia, as part of a real world global study, evaluated treatment patterns and associated survival outcomes in stage III NSCLC in Asia.

**Methods:**

Retrospective data from 57 centers in patients with stage III NSCLC diagnosed between January 2013 and December 2017 were analyzed. Median progression free survival (mPFS) and median overall survival (mOS) estimates with two sided 95% confidence interval (CI) were determined by applying the Kaplan-Meier survival analysis.

**Results:**

Of the total 1874 patients (median age: 63.0 years [24 to 92]) enrolled in the Asia subset, 74.8% were men, 54.7% had stage IIIA disease, 55.7% had adenocarcinoma, 34.3% had epidermal growth factor receptor mutations (EGFRm) and 50.3% had programmed death-ligand 1 (PD-L1) expression (i.e. PD-L1 ≥1%). Of the 31 treatment approaches as initial therapy, concurrent chemoradiotherapy (CRT) was the most frequent (29.3%), followed by chemotherapy (14.8%), sequential CRT (9.5%), and radiotherapy (8.5%). Targeted therapy alone was used in 81 patients of the overall population. For the Asia cohort, the mPFS and mOS were 12.8 months (95% CI, 12.2–13.7) and 42.3 months (95% CI, 38.1–46.8), respectively. Stage IIIA disease, Eastern Cooperative Oncology Group ≤1, age ≤65 years, adenocarcinoma histology and surgery/concurrent CRT as initial therapy correlated with better mOS (p < 0.05).

**Conclusions:**

The results demonstrate diverse treatment patterns and survival outcomes in the Asian region. The high prevalence of EGFRm and PD-L1 expression in stage III NSCLC in Asia suggests the need for expanding access to molecular testing for guiding treatment strategies with tyrosine kinase inhibitors and immunotherapies in this region.

## 1 Introduction

Lung cancer is amongst the most fatal cancers globally, accounting for 18% of all cancer deaths in 2020. About 59.6% of the world’s new lung cancer cases and 61.9% of lung cancer-related deaths occurred in Asia, in 2020 (1). Non-small cell lung cancer (NSCLC) accounts for approximately 85% of all lung cancer cases (2); about one-third (around 30%) of all NSCLC cases present with stage III (locally-advanced [LA]) disease (3, 4). The treatment choices for stage III NSCLC are primarily determined by tumor size, nodes and metastases staging, clinical presentation (patient’s age, performance status) and tumor pathology at initial diagnosis. According to the American Joint Committee on Cancer (AJCC) staging system (7th edition), stage III includes two subtypes, stage IIIA and IIIB (5). In 2017, stage IIIC was added to include LA T3 and T4 tumors associated with N3 disease but without metastasis for better prognostication (AJCC, 8^th^ edition) (6). The heterogeneous nature of stage III disease makes the management challenging and often warrants an integrative multidisciplinary decision for using a multimodal and personalized management approach (7). In the pre immuno-oncology (IO) era, curative surgery was the preferred treatment in a subset of stage IIIA disease, followed by chemotherapy (CT) (8). The National Comprehensive Cancer Network (NCCN) Clinical Practice Guidelines in Oncology (NCCN Guidelines^®^) April 2022 recommend osimertinib for patients with completely resected stage III epidermal growth factor receptor (EGFR) mutation positive NSCLC who received previous adjuvant CT or are ineligible to receive platinum based CT (9). In patients with microscopic residual disease, sequential chemoradiotherapy (sCRT) or concurrent chemoradiotherapy (cCRT) and in patients with macroscopic residual disease cCRT is the preferred treatment option (9). For patients with unresectable stage III disease, definitive cCRT (platinum-based doublet regimens), followed by durvalumab consolidation is recommended as a treatment option in patients who have not progressed after definitive cCRT (9). The treatment practices within Asia vary from country to country such as induction CT followed by radiotherapy (RT) in India (stage III/IV), surgery or neoadjuvant therapy or definitive chemoradiotherapy (CRT) in Korea (stage III) and cCRT in Singapore (stage III) (10–12). With a high prevalence of epidermal growth factor receptor mutations (EGFRm) in China (46.5%, 309/665), CT was followed by tyrosine kinase inhibitors (TKIs) in most (66.3%, 205/309) patients with unresectable stage IIIB/IV (13). Regional adaptations to international guidelines have also been developed (2, 14).

The survival outcomes reported for stage III NSCLC in Asia are generally poor with 5-year survival ranging from 3.4% to 34.9% (15–17). Hence, there is a need to understand the factors responsible for treatment decisions in the Asian region to recognize the unmet need to translate the newer treatment modalities into clinical practice in this region, with the objective of improving survival in this patient population. Databases or resources from Asian countries having information on diagnosis, treatment patterns and clinical outcomes for patients with stage III NSCLC are scarce. The recently published real-world KINDLE study was conducted internationally to characterize the treatment patterns and survival outcomes in the pre IO/pre TKI era for patients with stage III NSCLC (18). We report on the treatment patterns and associated survival outcomes of the Asia subset of the KINDLE study.

## 2 Materials and methods

### 2.1 Study design

KINDLE-Asia subset included eight countries (India, Indonesia, Korea, Malaysia, Singapore, Taiwan, Thailand and Vietnam) with 57 centers and enrolled consecutive patients diagnosed with *de novo* LA stage III NSCLC (AJCC 7th edition) between January 2013 and December 2017 with at least 9 months of documented follow up. The study was conducted in accordance with the Declaration of Helsinki, International Council for Harmonisation, good clinical practices, good pharmacoepidemiology practices and the other applicable regulations for noninterventional studies. The study protocol (NCT03725475) was reviewed and approved by the Institutional Review Boards/Independent Ethics Committees from all the participating centers before the initiation of the study. The reporting in this manuscript has been done following the Strengthening the Reporting of Observational Studies in Epidemiology checklist (19). The study eligibility criteria and data collection methods have been reported by Jazieh et al. (18) The study data (demography, clinical characteristics, treatment patterns and clinical outcomes) were collected retrospectively from patients’ medical records after obtaining written informed consent from the patients or their next of kin (in the case of deceased patients), or the legal representatives. The study outcomes are defined in **Supplementary Table S1**.

### 2.2 Statistical analyses

Patient demographics, clinical characteristics and treatment patterns were described using frequencies and percentages for categorical variables, mean/median and standard deviation with a 95% confidence interval (CI) as applicable for continuous variables. Median survival estimates (progression-free survival [PFS] and overall survival [OS]) were determined descriptively by applying the Kaplan-Meier survival analysis and log-rank test. A multivariate Cox proportional hazards model and hazards ratio (HR) along with 95% CI were used to identify the effects of clinical and demographic factors on OS and PFS by controlling relevant covariates affecting OS and PFS. A p value of less than 0.05 was considered statistically significant.

## 3 Results

### 3.1 Demographic and clinical characteristics

A total of 1874 patients were enrolled in the Asia subset with India (26%) and Korea (25%) combined contributing to around half of the study population. Detailed demographic and clinical characteristics are presented previously, as part of global data (18). The median age of the subset was 63.0 years (range: 24 to 92); 74.8% were men and 28.0% never smoked. At diagnosis, 54.7% of the patients had stage IIIA disease (AJCC, 7th edition) and 55.7% had adenocarcinoma. Of the patients with available data on Eastern Cooperative Oncology Group (ECOG) performance status, 88.9% had a performance status of ≤1. Surgical resection was performed in 23.3% (437/1874) (IIIA: 379; IIIB: 46) of the patients and 40.4% (758/1874) (IIIA: 320; IIIB: 417) had an unresectable disease. There were significant differences between resectable and unresectable patients in all clinical characteristics (all p<0.001) except for PD-L1 expression (**Supplementary Table S2**).

About one-third (600/1874, 32.0%) of the cases were discussed in the multidisciplinary team (MDT) meetings. Similar percentages of patients with stages IIIA and IIIB (34.8% and 30.2%) and those with resectable and unresectable diseases (33.4% and 31.7%) were discussed in MDT meetings (**Table 1**).

**Table 1:** Outcome discussed at the multidisciplinary team meeting in KINDLE-Asia.

Was the patient case discussed at an MDT meeting?	Asia (N = 1874)	Stage IIIA (N = 976)	Stage IIIB (N = 808)	Resectable (N = 437)	Unresectable (N = 758)
Yes, n (%)	600 (32.1)	339 (34.8)	244 (30.2)	146 (33.4)	240 (31.7)
No, n (%)	859 (46.0)	451 (46.3)	367 (45.5)	222 (50.8)	443 (58.4)
Unknown, n (%)	409 (21.9)	184 (18.9)	196 (24.3)	69 (15.8)	75 (9.9)

MDT, Multidisciplinary team; N, Number of patients; n, Number of patients in the subcategories.

### 3.2 Molecular testing

A total 865 (46.2%) patients underwent EGFRm testing at primary diagnosis, of whom 297 (34.3%) patients were found to have EGFRm in the Asia subset (**Supplementary Table S2**).

In stage IIIA disease, the percentage of patients undergoing a test for EGFRm was higher in resectable compared with unresectable patients (64.1% vs 40.3%) whereas, in stage IIIB, it was almost similar (52.2% vs 54.2%). The percentage of patients with of EGFRm was higher in resectable than in unresectable patients in stage IIIA disease (46.1% vs 30.2%); however, it was almost similar irrespective of resectability status in stage IIIB (25.0% vs 28.8%) (**Supplementary Table S3**).

The percentages of EGFRm were similar irrespective of gender (51.5% in females vs 48.5% in males) and resectability (52.9% in resectable vs unresectable in 47.1%) and were higher in never smokers than in current smokers (58.9% vs 11.4%) (**Supplementary Table S4**).

At primary diagnosis, testing for programmed death-ligand 1 (PD-L1) expression was performed for 292 (15.6%) patients of whom 147 (50.3%) tested positive for PD L1 (i.e. PD-L1 ≥1%) (**Supplementary Table S2**). The percentage of testing for PD-L1 expression was similar in both resectable and unresectable patients (21.6% vs 18.7%). In stage IIIA, a higher percentage of resectable than unresectable patients tested positive for PD L1 (52.9% vs 45.5%), whereas, in stage IIIB, higher percentage of patients with unresectable than the resectable disease (66.7% vs 57.1%) were positive for PD-L1 expression (**Supplementary Table S3**).

### 3.3 Treatment patterns

Overall, 94.5% (1771/1874) of the patients received an initial therapy (stage IIIA: 95.4% [931/976], stage IIIB: 94.8% [766/803]). cCRT-based therapies (34.3%) were used more frequently than curative surgery-based therapies (23.2%), systemic treatment (20.5%), RT-based (11.6%) and sCRT-based therapies (10.4%) (**Supplementary Table S5**). These categories included 31 different treatment approaches. The frequent approach used as the initial line was cCRT (29.3%), followed by CT (14.8%), sCRT (9.5%), RT (8.5%) and other surgeries such as surgery combined with neoadjuvant and/or adjuvant cCRT/CT/RT/sCRT/targeted therapy/IO drugs (6.5%). Post relapse, 746/1874 (39.8%) patients received second-line therapy and 282 (15.1%) of them received third-line therapy. In second- and third-line settings, CT was the predominant treatment (37.8% [282/746] and 36.9% [104/282]) followed by RT (18.9% [141/746] and 20.9% [59/282]) and targeted therapy alone (13.4% [100/746] and 11.0% [31/282]) in overall stage III population (**Supplementary Table S5** and **Figure S1**).

In stage IIIA, curative surgery-based treatment was the most common approach (37.5%) as initial treatment followed by cCRT-based therapies (30.2%), systemic treatment (13.6%), sCRT-based (9.3%) and RT-based therapies (9.3%). Whereas in stage IIIB, cCRT-based therapy was the most common approach (39.4%) as initial treatment followed by systemic treatment (29.0), RT based (13.2%), sCRT-based (11.5%) and curative surgery-based therapies (6.9%) (**Supplementary Table S5**).

Treatment pattern analyses as per resection status revealed that other surgery (22.2%), surgery+CT (20.0%) and surgery+sCRT (16.0%) were the top three treatments used in resectable patients (n=437) as initial-line treatment. The use of cCRT predominated (44.7%) in unresectable patients (n=758); the other frequent treatments were CT alone (15.2%), RT (11.8%), sCRT (8.9%) and targeted therapy alone (5.5%) (**Supplementary Figure S2** and **Table S6**).

In this unresectable category, when compared with patients receiving initial therapy with cCRT, a significantly higher percentage of patients receiving targeted therapy were females (50% vs 21.7%, p=0.0001), had stage IIIB disease (79.5% vs 51.9%, p=0.008), had adenocarcinoma histology (95%, vs 50.2%, p=0.002) and never smoked (67.5% vs 24.5%, p<0.001) (**Supplementary Table S7**).

### 3.4 Survival outcomes

**Figure 1:**
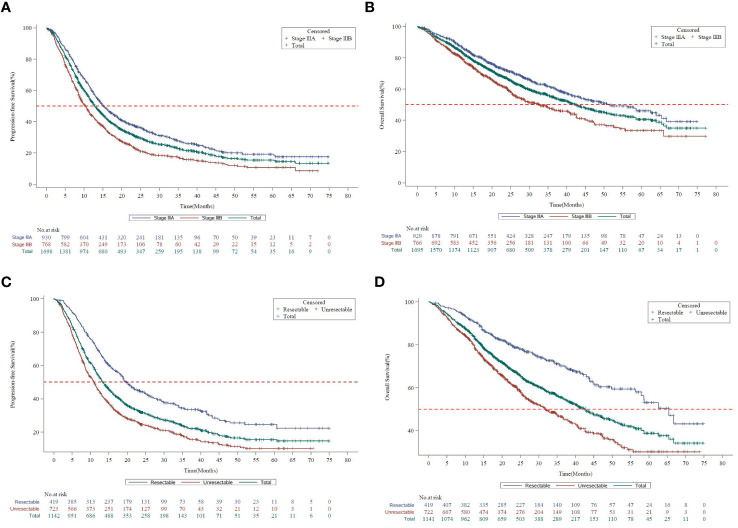
Survival curves by disease stage in KINDLE-Asia. **(A)** Kaplan-Meier survival curves for progression-free survival by disease stage (AJCC 7^th^ Edition). AJCC=American Joint Committee on Cancer; CI=Confidence interval; mPFS=Median progression-free survival; NSCLC=Non-small cell lung cancer. Kaplan-Meier Survival curves for progression-free survival for all stage III NSCLC patients are shown in green, whereas stage IIIA and stage IIIB patients are shown in blue or red, respectively. mPFS for the entire cohort, 12.8 months (95% CI, 12.19 to 13.70). mPFS for stage IIIA, 15.1 months (95% CI, 14.03 to 16.56). mPFS for stage IIIB, 10.3 months (95% CI, 9.26 to 11.27). **(B)** Kaplan-Meier survival curves for overall survival by disease stage (AJCC 7^th^ Edition). AJCC=American Joint Committee on Cancer; CI=Confidence interval; mOS=Median overall survival; NSCLC=Non-small cell lung cancer. Kaplan-Meier survival curves for overall survival for all stage III NSCLC patients are shown in green, whereas stage IIIA and stage IIIB patients are shown in blue or red, respectively. mOS for the entire cohort, 42.3 months (95% CI, 38.08 to 46.75). mOS for stage IIIA, 51.4 months (95% CI, 43.83 to 64.07). mOS for stage IIIB, 32.8 months (95% CI, 27.66 to 40.61). **(C)** Kaplan-Meier survival curves for progression-free survival by resection status. CI=Confidence interval; mPFS=Median progression-free survival; NSCLC=Non-small cell lung cancer. Kaplan-Meier survival curves for progression-free survival for all stage III NSCLC patients are shown in green, whereas resectable and unresectable patients are shown in blue or red, respectively. mPFS for the entire cohort, 12.8 months (95% CI, 12.19 to 13.70). mPFS for resectable patients, 19.8 months (95% CI, 18.00 to 22.67). mPFS for unresectable patients, 11.0 months (95% CI, 9.66 to 11.86) **(D)** Kaplan-Meier survival curves for overall survival by resection status. CI=Confidence interval; mOS=Median overall survival; NSCLC=Non-small cell lung cancer. Kaplan-Meier survival curves for overall survival for all stage III NSCLC patients are shown in green, whereas resectable and unresectable patients are shown in blue or red, respectively. mOS for the entire cohort, 42.3 months (95% CI, 38.08 to 46.75). mOS for resectable patients, 65.4 months (95% CI, 57.86 to Not Calculable). mOS for unresectable patients, 31.8 months (95% CI, 27.40 to 36.70).

In stage III NSCLC, the median progression-free survival (mPFS) and the median overall survival (mOS) for the Asia subset were 12.8 months (95% CI, 12.2 to 13.7) and 42.3 months (95% CI, 38.1 to 46.8), respectively. The mPFS and mOS were better for stage IIIA (15.1 months, 95% CI, 14.0 to 16.6 and 51.4 months, 95% CI, 43.8 to 64.1) than stage IIIB (10.3 months, 95% CI, 9.3 to 11.3 and 32.8 months, 95% CI, 27.7 to 40.6) (**Figures 1A, B**).

The mPFS (19.8 months vs 11.0 months) and mOS (65.4 months vs 31.8 months) were comparatively higher in patients with resectable than the unresectable disease (**Figures 1C, D**).

#### 3.4.1 Survival outcomes by initial treatment

The survival outcomes are presented as per the resection status and initial treatment. Amongst the top five treatments in the resectable category, surgery-based initial treatment followed by adjuvant treatment strategies in sequence showed better mPFS (29.9 months) than surgery alone (15.4 months) or CT alone (15.1 months), while mOS was better with CT alone (65.4 months) and surgery+CT (57.9 months) than surgery alone (32.1 months) (**Table 2** and **Supplementary Table S8**).

**Table 2:** Survival outcomes with top initial treatment patterns according to resection status and disease stage (AJCC 7^th^ Edition) in KINDLE-Asia.

2A. Per resection status										
S. No.	Treatment	Resectable months (95% CI)				Treatment	Unresectable months (95% CI)			
		N	mPFS	N	mOS		N	mPFS	N	mOS
1	Other surgery	93	29.9 (21.13-43.20)	93	NC (NC-NC)	cCRT	323	11.3 (9.40-13.04)	323	39.2 (32.36-50.79)
2	Surgery+CT	84	17.8 (12.06-25.03)	84	57.9 (42.94-NC)	CT	110	6.7 (5.91-8.71)	110	25.1 (17.31-42.61)
3	Surgery+sCRT	67	29.3 (18.00-NC)	67	NC (43.83-NC)	RT	85	10.4 (7.39-12.19)	85	16.8 (12.19-27.24)
4	CT	37	15.1 (6.74-23.72)	37	65.4 (43.83-NC)	sCRT	64	12.5 (9.43-14.95)	64	26.6 (18.56-36.70)
5	Surgery	33	15.4 (11.24-24.41)	33	32.1 (23.26-66.73)	Targeted therapy	40	13.8 (6.44-16.56)	40	24.0 (14.62-30.52)
2B. Per disease stage										
S. No	Treatment	Stage IIIA months (95% CI)				Treatment	Stage IIIB months (95% CI)			
		N	mPFS	N	mOS		N	mPFS	N	mOS
1	cCRT	247	14.4 (12.45-18.04)	247	50.8 (37.09-NC)	cCRT	254	9.3 (8.21-11.20)	254	36.0 (28.62-47.38)
2	CT	100	9.6 (6.64-12.48)	100	40.7 (29.24-65.38)	CT	150	7.4 (6.51-9.30)	149	24.2 (19.98-38.08)
3	Other surgery	93	26.7 (20.17-39.95)	93	NC (45.01-NC)	sCRT	78	9.4 (8.51-12.42)	78	25.7 (17.18-NC)
4	Surgery+CT	87	15.6 (11.66-21.91)	87	57.9 (37.82-NC)	RT	63	8.0 (4.60 -10.84)	63	13.0 (9.13-28.71)
5	sCRT	82	13.4 (10.74-14.95)	82	29.0 (26.05-NC)	Target therapy	58	10.5 (6.05-15.31)	58	27.7 (24.18-50.33)

AJCC, American Joint Committee on Cancer; cCRT, Concurrent chemoradiotherapy; CI, Confidence interval; CT, Chemotherapy; mOS, Median overall survival; mPFS, Median progression-free survival; N, Number of patients; NC, Not calculable; RT, Radiotherapy; sCRT, Sequential chemoradiotherapy.

The treatment pattern definitions based on the available patterns from the full analysis set for first line used until 1^st^ progressive disease.

IO: Immuno-oncology, Surgery+CT: surgery and chemotherapy were used in sequence, surgery+sCRT: surgery and sCRT were used in sequence, CT: only chemotherapy was used, Surgery: only surgery was used, Other Surgery: other therapies used in combination with surgery, cCRT: only cCRT was used, RT: only radiotherapy was used, Targeted therapy: only targeted therapy was used.

We found mPFS to be almost similar for all top five treatments used in unresectable category, except for CT alone; whereas mOS was better with cCRT (n=323, 39.2 months, 95% CI, 32.4 to 50.8) compared to sCRT (n=64, 26.6 months, 95% CI, 18.7 to 36.7, p=0.04), CT alone (n=110, 25.1 months, 95% CI, 17.3 to 42.6, p=0.02), targeted therapy alone (n=40, 24.0 months, 95% CI, 14.6 to 30.5, p=0.0006) or RT alone (n=85, 16.8 months, 95% CI, 12.2 to 27.2, p<0.0001) used until 1st progressive disease (**Table 2** and **Supplementary Tables S8**, **S9**).

Survival outcomes as per initial treatment according to AJCC staging (7th Edition) are described in **Table 2** and **Supplementary Table S10**.

In stage IIIA disease, amongst the top five treatments as initial treatments, other surgery showed better mPFS (n=93, 26.7 months) compared with cCRT (n=247,14.4 months), sCRT (n=82, 13.4 months) or CT alone (n=100, 9.6 months). While the mOS was better with surgery+CT (n=87, 57.9 months) than cCRT (n=247, 50.8 months), CT (n=100, 40.7 months) or sCRT (n=82, 29.0 months). In stage IIIB disease, the mPFS was almost similar for all top treatments, whereas mOS was better with cCRT (n=254, 36.0 months) compared with targeted therapy alone (n=58, 27.7 months), sCRT (n=78, 25.7 months) or CT alone (n=149, 24.2 months) (**Table 2** and **Supplementary Table S10**).

#### 3.4.2 Survival outcomes by EGFR mutation status

**Figure 2:**
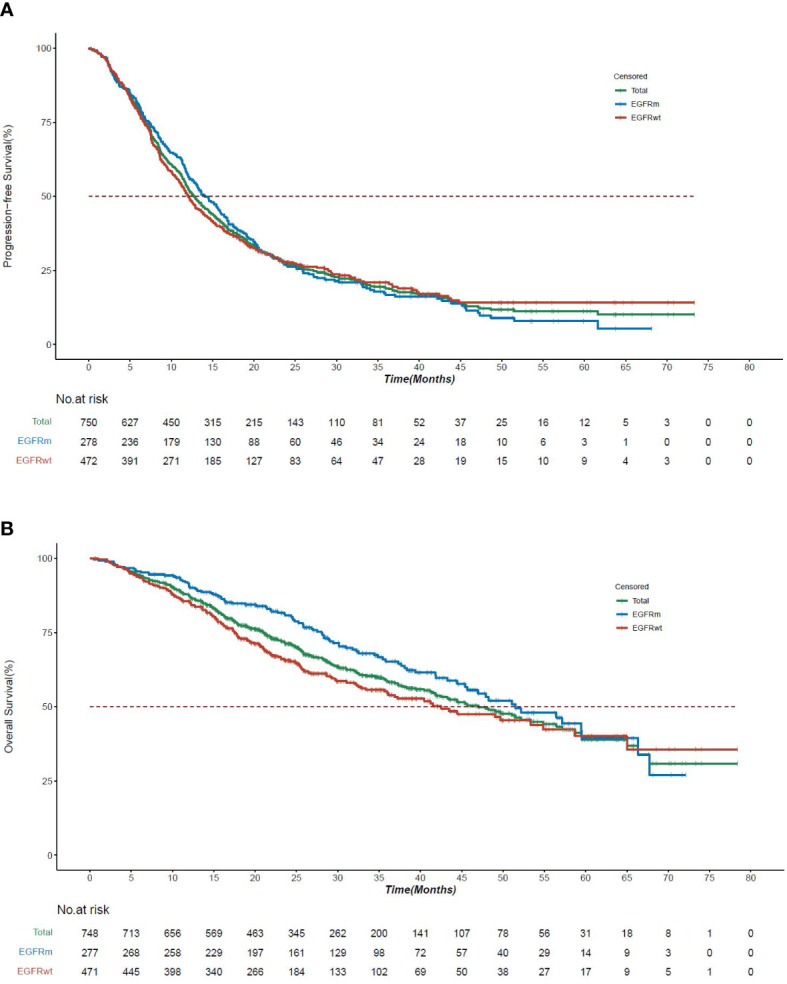
Survival curves by EGFR mutation status in KINDLE-Asia. **(A)** Kaplan-Meier survival curves for progression-free survival by EGFR mutation status. CI=Confidence interval; EGFR=Epidermal growth factor receptor; EGFRm=Epidermal growth factor receptor mutation; EGFRwt=Epidermal growth factor receptor wild type mutation; mPFS=Median progression-free survival; NSCLC=Non-small cell lung cancer. Kaplan-Meier survival curves for progression-free survival for all stage III NSCLC patients are shown in green, whereas EGFRm and EGFRwt patients are shown in blue or red, respectively. mPFS for the entire cohort, 12.8 months (95% CI, 12.19 to 13.70). mPFS for EGFRm patients, 14.1 months (95% CI, 12.6 to 16.4). mPFS for EGFRwt patients, 12.0 months (95% CI, 11.1 to 13.6). **(B)** Kaplan-Meier survival curves for overall survival by EGFR mutation status. CI=Confidence interval; EGFR=Epidermal growth factor receptor; EGFRm=Epidermal growth factor receptor mutation; *EGFR*wt=Epidermal growth factor receptor wild type mutation; mOS=Median overall survival; NSCLC=Non-small cell lung cancer. Kaplan-Meier survival curves for overall survival for all stage III NSCLC patients are shown in green, whereas EGFRm and EGFRwt patients are shown in blue or red, respectively. mOS for the entire cohort, 42.3 months (95% CI, 38.08 to 46.75). mOS for EGFRm patients, 51.5 months (95% CI, 45.4 to 67.7). mOS for EGFRwt patients, 42.5 months (95% CI, 35.7 to 58.7).

The mPFS and mOS for patients with EGFRm were 14.1 months (95% CI, 12.6 to 16.4) and 51.5 months (95% CI, 45.4 to 67.7), respectively, which were longer than patients not having EGFRm (**Figures 2A, B**). In patients with EGFRm having resectable disease, the mPFS and mOS were longer (19.1 months, 59.5 months) compared to patients with the unresectable disease (13.2 months, 48.2 months) (**Supplementary Table S11**).

The use of targeted therapy was more frequent as initial therapy in patients with EGFRm (61/297, 20.5%); the mPFS and mOS for these patients were 11.2 months (n=61, 95% CI, 7.16 to14.3) and 25.4 months (n=61, 95% CI, 21.6 to 34.9). The other preferred treatment options in EGFR mutated patients were cCRT (43/297, 14.5%); the mPFS and mOS for these patients were 11.5 months (95% CI, 6.05 to 16.16) and 50.8 months (95% CI, 47.21 to not calculable [NC]), respectively (**Supplementary Figure S3** and **Table 3**).

**Table 3:** Survival outcomes with top initial treatment patterns according to *EGFR* mutation status in KINDLE-Asia.

			EGFRm					EGFRwt		
S. No.	Treatment	N	mPFS months (95% CI)	N	mOS months (95% CI)	Treatment	N	mPFS months (95% CI)	N	mOS months (95% CI)
1	Target therapy	61	11.2 (7.16-14.29)	61	25.4 (21.62 34.92)	cCRT	139	9.5 (8.41-12.29)	139	40.6 (25.59-64.07)
2	cCRT	43	11.5 (6.05-16.16)	43	50.8 (47.21-NC)	CT	96	7.4 (5.91-10.32)	95	29.2 (20.44-NC)
3	Other surgery	31	25.6 (16.66-41.59)	31	NC (31.31-NC)	Other surgery	37	28.1 (16.07-NC)	37	NC (35.61- NC)
4	Surgery+CT	23	13.0 (8.87- 28.19)	23	58.6 (37.82- NC)	Surgery+CT	36	15.6 (12.06-20.67)	36	29.4 (21.13-57.86)
5	CT	23	15.4 (6.67-19.02)	23	NC (65.38-NC)	sCRT	32	12.6 (8.48-16.99)	32	36.7 (17.31 to NC)

cCRT, Concurrent chemoradiotherapy; CI, Confidence interval; CT, Chemotherapy; EGFRm, Epidermal growth factor receptor mutation; EGFRwt, Epidermal growth factor receptor wild type mutation; mOS, Median overall survival; mPFS, Median progression-free survival; N, Number of patients; NC, Not calculable; RT, Radiotherapy; sCRT, Sequential chemoradiotherapy.

The treatment pattern definitions based on the available patterns from the full analysis set for first line used until 1^st^ progressive disease

IO: Immuno-oncology, Surgery+CT: surgery and chemotherapy were used in sequence, surgery+sCRT: surgery and sCRT were used in sequence, CT: only chemotherapy was used, Surgery: only surgery was used, Other Surgery: other therapies used in combination with surgery, cCRT: only cCRT was used, RT: only radiotherapy was used, Targeted therapy: only targeted therapy was used.

### 3.5 Prognostic factors of mPFS and mOS

Clinical and demographic prognostic factors for mPFS and mOS for the overall population (**Table 4**) were assessed using univariate and multivariate analyses.

**Table 4:** Univariate and multivariate analyses for survival outcomes in KINDLE-Asia.

Characteristics	Univariate analyses					
	PFS			OS		
	N	HR (95% CI)	p-value	N	HR (95% CI)	p-value
Stage IIIA vs IIIB	930 vs 768	0.671 (0.600-0.750)	**<0.0001**	929 vs 766	0.659 (0.566-0.768)	**<0.0001**
Age >65 vs ≤65	717 vs 1055	1.156 (1.035-1.291)	**0.0103**	717 vs 1051	1.345 (1.157-1.563)	**0.0001**
ECOG 0/1 vs 2/3/4	989 vs 124	0.688 (0.559-0.849)	**0.0005**	985 vs 124	0.533 (0.409-0.696)	**<0.0001**
EGFRm vs EGFRwt	281 vs 488	1.008 (0.855-1.188)	0.9221	280 vs 487	0.723 (0.568-0.920)	**0.0082**
Male vs female	1320 vs 452	1.026 (0.906-1.162)	0.6847	1316 vs 452	1.542 (1.284-1.853)	**<0.0001**
Smoking history yes vs no	1096 vs 504	1.109 (0.980-1.255)	0.1022	1094 vs 502	1.534 (1.288-1.826)	**<0.0001**
Resectable yes vs no	419 vs 723	0.553 (0.478-0.640)	**<0.0001**	419 vs 722	0.477 (0.388-0.585)	**<0.0001**
Adenocarcinoma vs others	983 vs 786	0.967 (0.866-1.080)	0.5531	980 vs 785	0.635 (0.546-0.737)	**<0.0001**
Surgery in initial treatment yes vs no	410 vs 1362	0.510 (0.443-0.586)	**<0.0001**	410 vs 1358	0.513 (0.422-0.624)	**<0.0001**
cCRT as initial treatment yes vs no	519 vs 1253	1.005 (0.891-1.134)	0.9349	519 vs 1249	0.940 (0.796-1.109)	0.4617
cCRT as initial treatment vs sCRT as initial treatment	519 vs 169	0.868 (0.711-1.058)	0.1616	519 vs 168	0.705 (0.541-0.920)	**0.0100**
Trimodality as initial treatment yes vs no	142 vs 1630	0.541 (0.432-0.677)	**<0.0001**	142 vs 1626	0.511 (0.367-0.712)	**<0.0001**
Characteristics	Multivariate analyses					
	PFS			OS		
	N	HR (95% CI)	p-value	N	HR (95% CI)	p-value
Stage IIIA vs IIIB	538 vs 458	0.779 (0.668-0.908)	**0.0014**	537 vs 456	0.709 (0.577-0.870)	**0.0010**
Age >65 vs ≤65	413 vs 583	1.085 (0.936-1.258)	0.2805	413 vs 580	1.304 (1.073-1.585)	**0.0076**
ECOG 0/1 vs 2/3/4	897 vs 99	0.752 (0.598-0.945)	**0.0147**	894 vs 99	0.584 (0.441-0.775)	**0.0002**
Male vs female	745 vs 251	0.962 (0.757-1.222)	0.7494	742 vs 251	1.140 (0.823-1.580)	0.4300
Smoking history yes vs no	685 vs 311	1.288 (1.027-1.615)	**0.0283**	684 vs 309	1.253 (0.926-1.696)	0.1438
Adenocarcinoma vs others	554 vs 442	1.140 (0.975-1.333)	0.1010	551 vs 442	0.809 (0.658-0.995)	**0.0451**
Surgery in initial treatment yes vs no	217 vs 779	0.504 (0.392-0.649)	**<0.0001**	217 vs 776	0.642 (0.463-0.891)	**0.0080**
cCRT as initial treatment yes vs no	335 vs 661	0.745 (0.632-0.878)	**0.0004**	335 vs 658	0.694 (0.558-0.864)	**0.0011**
Trimodality as initial treatment yes vs no	85 vs 911	0.902 (0.629-1.293)	0.5755	85 vs 908	0.807 (0.487-1.339)	0.4070

AJCC, American Joint Committee on Cancer; cCRT, Concurrent chemoradiotherapy; CI, Confidence interval; ECOG, Eastern Cooperative Oncology Group; EGFRm, Epidermal growth factor receptor mutation; EGFRwt, Epidermal growth factor receptor wild type mutation; HR, Hazard ratio; N, Number of patients; OS, overall survival; PFS, Progression-free survival; sCRT, Sequential chemoradiotherapy.

Stage of tumor is per AJCC 7^th^ edition.

Values in bold indicate significant difference (p<0.05).

In the overall stage III population, univariate analyses showed significantly longer mPFS and mOS in patients with stage IIIA disease, aged ≤65 years, with ECOG ≤1, with resected disease and having undergone surgery or received triple therapy as initial treatment (p < 0.05 for all). Additionally, EGFRm, female gender, no smoking history, adenocarcinoma and having received cCRT as part of initial treatment predicted longer mOS (p < 0.05 for all).

In multivariate analyses, stage IIIA disease, ECOG ≤1, and surgery or cCRT as part of initial therapy were independently associated with better mPFS and mOS in the overall stage III population (p<0.05 for all). Age ≤65 years and adenocarcinoma were additional independent predictors of better mOS (p<0.05 each). Whereas no smoking history was independently associated with better mPFS (p<0.05).

Further, the predictors associated with stage IIIA and IIIB disease are shown in **Supplementary Tables S12**, **S13** present.

## 4 Discussion

We present the multinational retrospective data from Asia on treatment practices and survival outcomes for stage III NSCLC patients, as a subset of the KINDLE study. Asian patients were predominantly older (>60 years) males. We found a higher percentage of patients in Asia who never smoked (28%) compared to other regions of the KINDLE study (Latin America, 14.8% and the Middle East and Africa, 16%) (18). The treatment diversity, with the use of about 31 approaches, indicates challenges posed by the heterogeneity of stage III disease and optimization of the treatment decision-making process in Asia.

As initial therapy, the most frequent treatment approach for the entire Asia subset (overall, stages IIIA and IIIB) was cCRT (29.3%, 26.5% and 33.2%) followed by CT alone (14.8%, 10.7% and 19.6%). These findings are in line with KINDLE-Global results (18). Because the majority of the patients had unresectable NSCLC, the choice of cCRT as the predominant initial therapy was appropriate as per the contemporary guidelines (20). In the second and third lines, CT alone was the most preferred treatment option. Unlike our findings, the predominant treatment patterns observed in other Asian real-word studies were curative intent surgery in Korea (49.6%) (10), platinum-based CT in Japan (56.0%) (21) and cCRT in Singapore (31.2%) (11). Our study provides more recent insights on treatment patterns in stage III NSCLC from the Asian countries compared with these studies. With changing treatment paradigm, more empirical studies are required from this region to explore patient, social and economic factors affecting the selection of treatment approaches including insurance coverage, accessibility and availability of newer targeted drugs.

The mPFS observed in the Asian population with stage III disease was 12.8 months, which is similar to the KINDLE-Global results (18) whereas, the mOS of 42.3 months is higher than the global cohort (34.9 months) (18). The mOS according to resectability and staging observed in our Asia subset were longer (in unresectable: 31.8 months; stage IIIB: 32.8 months) than other large-scale real-world studies from the United States in unresected stage III NSCLC (mOS: 20 months) (22), and Portugal (mOS: 11.4 months in stage IIIB disease) (23). We found an independent association between longer mOS and stage IIIA disease, ECOG ≤1, age ≤65 years, adenocarcinoma histology, and surgery or cCRT as initial therapy. Similarly, other real world studies have reported an association between decent ECOG performance status, younger age, early-stage disease, cCRT or surgery as a part of initial treatment and a lesser risk of death in patients with NSCLC (22, 24). In our cohort, we also noted an association between EGFRm and better mOS (HR: 0.723, 95% CI, 0.568 to 0.920, p=0.0082). The role of higher prevalence of EGFRm in deciding subsequent treatment choices and better survival in Asian population needs further exploration.

In a Korean study in stage III NSCLC, the mOS was highest for curative-intent surgery (52.5 months, 95% CI, 43.1 to 61.9), and 49.2 months (95% CI, 42.0 to 56.5) in those who received neoadjuvant therapy (10). We report similar OS benefits in stage IIIA patients receiving surgery based treatments such as surgery+CT (57.9 months, 95% CI, 37.8 to NC) or surgery+RT (58.6 months, 95% CI, 14.5 to NC). In unresectable patients, cCRT significantly improved OS compared with sCRT, CT alone or RT alone. These findings resonate with significantly improved survival outcomes reported with cCRT than sCRT (HR: 0.84; p=0.004) (25), CT alone and RT alone in a systematic review and meta analyses and in a few other single-center studies (26–28).

The role of a MDT in treatment decision-making is well established and augments patient outcomes (29–32). The MDT was involved in treatment decisions for only one third of the cases (32.0%) in this study. Considering the upcoming molecular and immunology testing-based novel modalities, active involvement of MDT needs to be encouraged in Asia for patient-centric management of stage III NSCLC.

The advent of immunotherapy and TKIs have changed the treatment paradigm of NSCLC over the past few years. Studies have shown that multimodal regimens using molecular targeting and/or immunotherapy provide survival benefits (33–36), leading to change in NCCN^®^ Guidelines (9) incorporating durvalumab as consolidation post CRT and adjuvant osimertinib post-surgery with or without platinum-based CT in the management of resectable stage III NSCLC. In Asian patients with NSCLC, the prevalence of EGFRm is high compared to the Western population (50% vs 15%) (37). Yang et al. reported an overall EGFRm rate of 51.4% in NSCLC stage IIIB/IV adenocarcinoma in the Asia region (range: 22.2% to 64.2%) (38). The KINDLE-Asia subset showed a higher EGFRm rate (34.3%) in stage III NSCLC, than other KINDLE regions (Middle East and Africa, 20.0% and Latin America, 28.4%) (39). EGFRm were more frequently found in females (51.5%), never smokers (58.9%), stage IIIA (62.2%), those with adenocarcinoma histology (92.3%) and resectable disease (52.9%). At primary diagnosis, a higher percentage of EGFR-mutated patients in our study had resectable tumors compared with patients without EGFRm (52.9% vs 37.3%). Results of the ADAURA phase III study demonstrated a clinically meaningful and significant improvement in disease-free survival with osimertinib in patients with NSCLC stage II-IIIA with EGFRm compared to placebo (HR: 0.17; 99.06% CI, 0.11 to 0.26, p<0.001) (33). Osimertinib reduced the risk of disease recurrence or death by 83%. In the overall study population of patients with stage IB-IIIA disease and EGFRm, the risk of disease recurrence or death was reduced by 80% (HR: 0.20, 99.12% CI, 0.14 to 0.30; p<0.001) (33). The updated 2022 NCCN guidelines recommend molecular testing for EGFRm to assess whether adjuvant TKI therapy could be an option for resectable stage IB IIIA NSCLC (9). The guidelines further recommend osimertinib for patients with completely resected stage IB-IIIA EGFRm-positive (exon 19 deletion, L858R) NSCLC, who received previous adjuvant CT or are ineligible to receive platinum-based CT (9). Furthermore, the ongoing LAURA phase III trial (NCT03521154) which is evaluating the role of osimertinib as maintenance therapy in patients with unresectable stage III NSCLC with EGFRm following cCRT will provide important evidence if EGFR-targeted therapy is beneficial for survival gain in unresectable stage III NSCLC with EGFR-mutated patients (40). In the background of this evolving evidence, treating oncologists should encourage genomic profiling in stage III NSCLC; in cases of resected patients, biopsied or resected samples are routinely sent for biomarker testing to plan further course of treatment; however, in unresectable patients, genomic profiling is delayed until progression to stage IV, when a liquid biopsy is a recommended option for planning targeted therapy (41).

In our study, in unresectable disease, cCRT was used in about one-third of the study population (in line with NSCLC management guidelines) and provided better mPFS (11.3 months) and mOS (39.2 months) than CT or RT alone; however, the remaining patients received CT alone, sCRT and RT alone with poor survival. Now, with durvalumab being approved, this group of unresectable stage III NSCLC patients would most likely benefit from durvalumab consolidation post cCRT (42), if early PD-L1 testing is encouraged. The 5-year OS data from the PACIFIC study demonstrated robust and sustained OS plus durable PFS benefit with the PACIFIC regimen with 42.9% of patients being alive and approximately 33% of the patients remained alive and free of disease progression (43). A retrospective study found that in clinical practice, approximately 70% of patients with unresectable stage III NSCLC not progressing on cCRT would be eligible to receive consolidation therapy with durvalumab (44).

The current findings from this Asia subset provide a benchmark to understand the existing treatment landscape, which will be important for implementing newer therapies and evaluating their effectiveness in this population. Though the study provides insights into treatment practices for stage III NSCLC in the Asian region, the retrospective design may limit the representativeness of the findings before immunotherapy approval. Being a real-word study, the data collection was limited to clinicians’ reports from the existing medical records and the data captured included data pertaining to the protocol-defined outcomes only. The details of histopathology (including pathologic confirmation of N2 lymph nodes) and other diagnostic work-up were not captured; which might have resulted in missing information about diagnostic practices. Some patients might have been lost to routine clinical follow-up, thus resulting in missing data. Additionally, retrospective data collection may have favored patients with longer survival, resulting in a potential bias in the study outcomes.

## 5 Conclusions

The results from this large, real-world study demonstrate diverse treatment patterns and survival outcomes in the Asian region, providing baseline data for evaluating novel therapies for stage III NSCLC in the near future. Nearly 31 treatment approaches were used with around 32% of the cases being discussed in MDT meetings. In unresectable disease, cCRT as initial therapy showed longer survival benefits than sCRT, RT alone, CT and targeted therapy. Surgery followed by adjuvant CT in resectable disease showed longer survival benefit than surgery alone. However, our findings also demonstrate limited adherence to the treatment guidelines applicable before immunotherapy approval including treatment decisions based on MDT discussions. The EGFRm testing rate of 46.2% in the overall stage III population and EGFRm positivity reported as 44.2% and 29.3% in resectable and unresectable categories, respectively, suggests the need for expanding access to molecular testing for guiding treatment strategies with TKIs and immunotherapies in the Asian region.

## Data availability statement

The original contributions presented in the study are included in the article/**Supplementary Material**. Further inquiries can be directed to the corresponding author.

## Ethics statement

The study protocol (NCT03725475) was reviewed and approved by the Institutional Review Boards/Independent Ethics Committees from all the participating centers before the initiation of the study: The Institutional Ethics Committees (IECs) Tata Memorial Centre (TMC), Mumbai; The SingHealth Centralised Institutional Review Board (CIRB), Singapore; The Domain Specific Review Board (DSRB), Singapore; The Institutional Review Board of the Faculty of Medicine at Chulalongkorn University, Thailand; Institutional Review Board of Taipei Veterans General Hospital, Taiwan; Medical Research and Ethics Committee (MREC), National Institute of Health, Malaysia; Persahabatan Hospital Ethic Committee, Indonesia; Yonsei University Health System, Severance hospital, Institutional review board, Republic of Korea. Written informed consent was obtained from the patients or their next of kin/legal representatives (in the case where patients were deceased) before retrospective data were collected.

## Author contributions

KP: Conceptualization, Methodology, Investigation, Writing, Review, Editing, Visualization, Validation. DT: Conceptualization, Investigation, Methodology, Review, Editing. RS: Conceptualization, Investigation, Methodology, Review, Editing. PS: Conceptualization, Investigation, Methodology, Review, Editing. YC: Conceptualization, Investigation, Methodology, Review, Editing. PV: Conceptualization, Investigation, Methodology, Review, Editing. ES: Conceptualization, Investigation, Methodology, Review, Editing. SC: Conceptualization, Investigation, Methodology, Review, Editing. RH: Conceptualization, Investigation, Methodology, Review, Editing. B-CC: Conceptualization, Methodology, Investigation, Writing, Review, Editing, Visualization, Validation. The work reported in the paper has been performed by the authors, unless clearly specified in the text. All authors contributed to the article and approved the submitted version.

## Supplementary material

Supplementary Figure S1 Frequent treatment patterns used in various lines of therapy for stage III NSCLC in KINDLE-Asia. cCRT, Concurrent chemoradiotherapy; CT, Chemotherapy; NSCLC, Non-small cell lung cancer; RT, Radiotherapy; sCRT, Sequential chemoradiotherapy. The treatment pattern definitions are based on the available patterns from the full analysis set for first line used until 1^st^ progressive disease. Other Surgery: other therapies used in combination with surgery, cCRT: only cCRT was used, sCRT: only sCRT was used, CT: only chemotherapy was used, IO: only immunotherapy was used, RT: only radiotherapy was used, Targeted therapy: only targeted therapy was used.

Supplementary Figure S2 Frequent initial treatment patterns according to disease stage (AJCC 7^th^ Edition) and resection status in KINDLE‑Asia. AJCC, American Joint Committee on Cancer; cCRT, Concurrent chemoradiotherapy; CT, Chemotherapy; IO, immune-oncology; RT, Radiotherapy; sCRT, Sequential chemoradiotherapy. The treatment pattern definitions are based on the available patterns from the full analysis set for first line used until 1st progressive disease. Surgery alone: only surgery was used, Surgery+sCRT: surgery and sCRT were used in sequence, Surgery+CT: surgery and chemotherapy were used in sequence, Other Surgery: other therapies used in combination with surgery, cCRT: only cCRT was used, sCRT: only sCRT was used, CT: only chemotherapy was used, RT: only radiotherapy was used, Targeted therapy: only targeted therapy was used

Supplementary Figure S3 Frequent initial treatment patterns according to *EGFR* mutation status. cCRT, Concurrent chemoradiotherapy; CT, Chemotherapy; *EGFR*, Epidermal growth factor receptor; IO, Immuno-oncology; RT, Radiotherapy; sCRT, Sequential chemoradiotherapy. The treatment pattern definitions are based on the available patterns from the full analysis set for first line used until 1^st^ progressive disease. Surgery+sCRT: surgery and sCRT were used in sequence, Surgery+CT alone: surgery and chemotherapy were used in sequence, cCRT: only cCRT was used, CT alone: only chemotherapy was used, Targeted therapy alone: only targeted therapy was used.
