# Supplementary figures and images for: Real-world clinical practice and outcomes in treating stage III non-small cell lung cancer: KINDLE-Asia subset
Source: Front Oncol. 2023 Mar 27;13:1117348. doi: 10.3389/fonc.2023.1117348 (PMC10083698; doi:10.3389/fonc.2023.1117348)

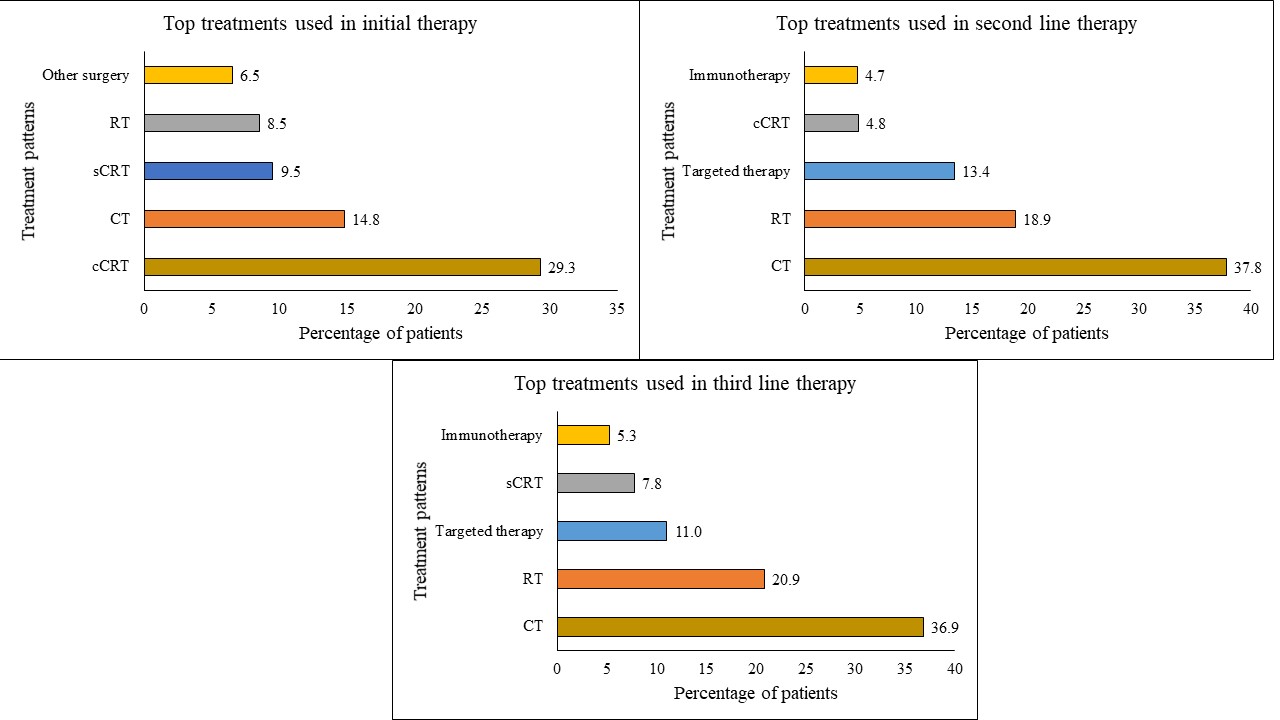

Supplement: Supplementary Figure S1 — Frequent treatment patterns used in various lines of therapy for stage III NSCLC in KINDLE-Asia. cCRT, Concurrent chemoradiotherapy; CT, Chemotherapy; NSCLC, Non-small cell lung cancer; RT, Radiotherapy; sCRT, Sequential chemoradiotherapy. The treatment pattern definitions are based on the available patterns from the full analysis set for first line used until 1st progressive disease. Other Surgery: other therapies used in combination with surgery, cCRT: only cCRT was used, sCRT: only sCRT was used, CT: only chemotherapy was used, IO: only immunotherapy was used, RT: only radiotherapy was used, Targeted therapy: only targeted therapy was used. [file Image_1.jpeg]

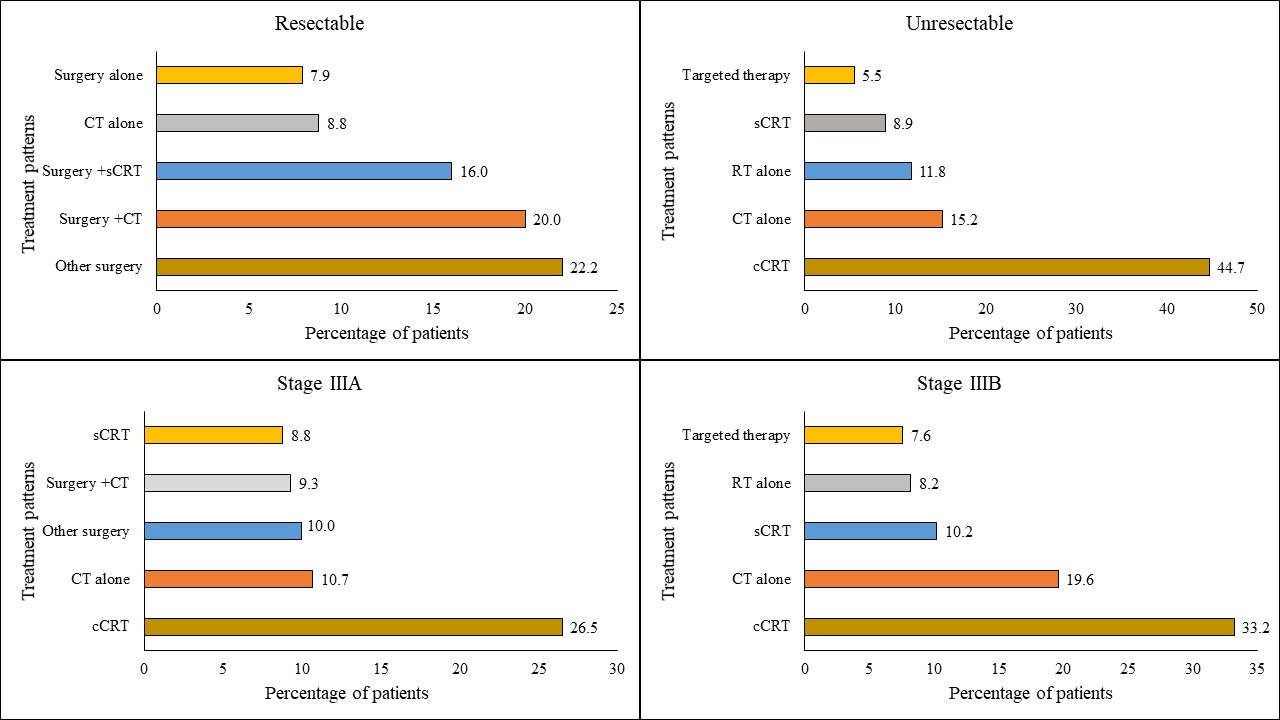

Supplement: Supplementary Figure S2 — Frequent initial treatment patterns according to disease stage (AJCC 7th Edition) and resection status in KINDLE‑Asia. AJCC, American Joint Committee on Cancer; cCRT, Concurrent chemoradiotherapy; CT, Chemotherapy; IO, immune-oncology; RT, Radiotherapy; sCRT, Sequential chemoradiotherapy. The treatment pattern definitions are based on the available patterns from the full analysis set for first line used until 1st progressive disease. Surgery alone: only surgery was used, Surgery+sCRT: surgery and sCRT were used in sequence, Surgery+CT: surgery and chemotherapy were used in sequence, Other Surgery: other therapies used in combination with surgery, cCRT: only cCRT was used, sCRT: only sCRT was used, CT: only chemotherapy was used, RT: only radiotherapy was used, Targeted therapy: only targeted therapy was used [file Image_2.jpeg]

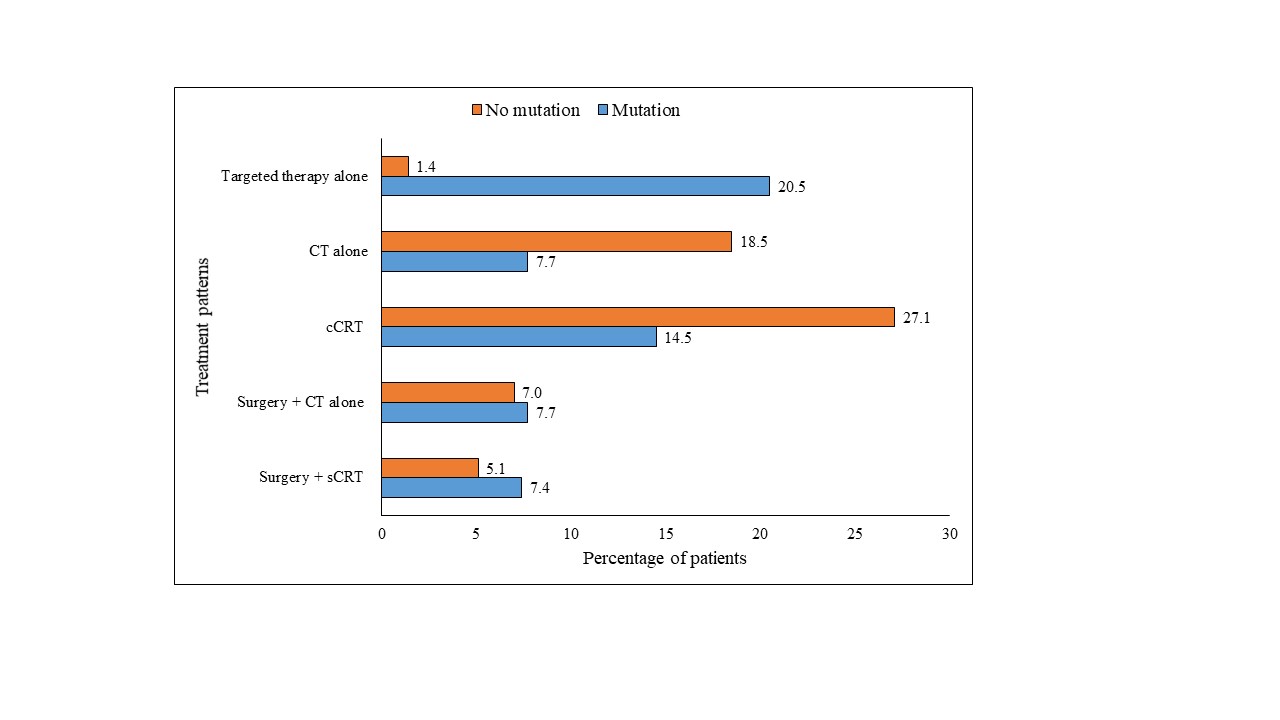

Supplement: Supplementary Figure S3 — Frequent initial treatment patterns according to EGFR mutation status. cCRT, Concurrent chemoradiotherapy; CT, Chemotherapy; EGFR, Epidermal growth factor receptor; IO, Immuno-oncology; RT, Radiotherapy; sCRT, Sequential chemoradiotherapy. The treatment pattern definitions are based on the available patterns from the full analysis set for first line used until 1st progressive disease. Surgery+sCRT: surgery and sCRT were used in sequence, Surgery+CT alone: surgery and chemotherapy were used in sequence, cCRT: only cCRT was used, CT alone: only chemotherapy was used, Targeted therapy alone: only targeted therapy was used. [file Image_3.jpeg]
