# Supplementary material for: Real-world clinical practice and outcomes in treating stage III non-small cell lung cancer: KINDLE-Asia subset
Source: Front Oncol. 2023 Mar 27;13:1117348. doi: 10.3389/fonc.2023.1117348 (PMC10083698; doi:10.3389/fonc.2023.1117348)
Supplement: Supplementary file 4 [file Table_1.docx]

Supplementary Material

Supplementary Tables

**Supplementary Table S1.** Study outcomes

| **Outcomes** | **Definition** | **Calculation** |
| --- | --- | --- |
| Overall survival | The length of time from stage III NSCLC diagnosis (index date) or time of therapy initiation to death due to any cause. | OS will be calculated from index date to date of death.  OS will also be calculated from the start of each LOT to date of death.  If patients are confirmed to be alive at the last record available/end of the observation period (date of data abstraction), time‑to‑event will be censored as of this date.  For deceased patients with unknown date of death and patients with unknown vital statuses, OS will be censored at the date of last record where the patient was known to be alive. As far as possible, information on the date of death will be collected from the primary care physician or from the next of kin/legal representative.  OS will be described overall and by subgroups of interest (if data permits). |
| Progression‑free survival | The length of time from time of therapy initiation to documented disease progression (as available in the medical record) or death due to any cause, whichever occurs first. | PFS will be calculated from the date of start of LOT to the date of the following documented disease progression or death, whichever occurs first.  For patients with no disease progression documented, PFS will be censored at the earliest at last medical record entry or end of the observation period (date of data abstraction).  Disease progression will be considered present if best response to treatment is PD; and absent if best response to treatment is CR, PR, SD or NE.  PFS will be described overall and by subgroups of interest (if data permits). |

CR=Complete response; LOT=Line of therapy; NE=Not evaluated; NSCLC=Non-small cell lung cancer; OS=Overall survival; PD=Progressive disease; PFS=Progression‑free survival; PR=Partial response; SD=Stable disease.

**Supplementary Table S2.** Clinical characteristics of stage III NSCLC and comparison by resectability status in KINDLE-Asia

| **Parameters** | **Asia** (N=1874) | **Comparison by resectability (N=1195)** | | |
| --- | --- | --- | --- | --- |
|  |  | **Resectable**^b^ (N= 437) | **Unresectable**^b^ (N=758) | **p-value** |
| County‑wise patient disposition n (%) |  | | | <0.001 |
| India | 494 | 50 (11.4) | 52 (6.9) |  |
| Indonesia | 78 | 8 (1.8) | 2 (0.3) |  |
| Malaysia | 81 | 15 (3.4) | 25 (3.3) |  |
| Thailand | 200 | 48 (11.0) | 125 (16.5) |  |
| Taiwan | 200 | 56 (12.8) | 125 (16.5) |  |
| Korea | 461 | 193 (44.2) | 244 (32.2) |  |
| Singapore | 210 | 49 (11.2) | 87 (11.5) |  |
| Vietnam | 150 | 18 (4.1) | 98 (12.9) |  |
| Age (years), median (range) | 63.0 (24-92) | 62.0 (32-87) | 65.0 (24-92) | <0.001 |
| Gender n (%) | | | | <0.001 |
| Female | 473 (25.2) | 157 (35.9) | 178 (23.5) |  |
| Male | 1401 (74.8) | 280 (64.1) | 580 (76.5) |  |
| BMI (kg/m^2^), median (range) | 22.5 (13-65) | 23.5 (15-65) | 22.2 (13-43) | <0.001 |
| Patient status, n (%) | | | | <0.001 |
| Alive | 1142 (60.9) | 305 (69.8) | 388 (51.2) |  |
| Dead | 732 (39.1) | 132 (30.2) | 370 (48.8) |  |
| Smoking Status^a^, n (%) | | | | <0.001 |
| Current smoker | 508 (27.1) | 109 (24.9) | 245 (32.3) |  |
| Ex-smoker | 653 (34.9) | 127 (29.1) | 262 (34.6) |  |
| Never smoker | 524 (28.0) | 169 (38.7) | 217 (28.6) |  |
| Disease stage (AJCC 7^th^ edition), n (%) | | | | <0.001 |
| Stage IIIA | 976 (54.7) | 379 (89.2) | 320 (43.4) |  |
| Stage IIIB | 808 (45.3) | 46 (10.8) | 417 (56.6) |  |
| Histology type, n (%) | | | | <0.001 |
| Adenocarcinoma | 1039 (55.7) | 310 (71.3) | 392 (51.8) |  |
| Epidermoid or squamous cell carcinoma | 648 (34.7) | 97 (22.3) | 278 (36.7) |  |
| Other^c^ | 76 (4.1) | 12 (2.8) | 44 (5.8) |  |
| Large cell carcinoma | 24 (1.3) | 10 (2.3) | 13 (1.7) |  |
| Mixed | 19 (1.0) | 4 (0.9) | 8 (1.1) |  |
| Bronchiole-alveolar | 3 (0.2) | 0 | 0 |  |
| ECOG performance status, n (%) | | | | <0.001 |
| 0 | 295 (25.5) | 111 (47.4) | 130 (26.2) |  |
| 1 | 735 (63.4) | 115 (49.2) | 300 (60.4) |  |
| 2 | 105 (9.1) | 8 (3.4) | 54 (10.9) |  |
| 3 | 21 (1.8) | 0 | 12 (2.4) |  |
| 4 | 3 (0.3) | 0 | 1 (0.2) |  |
| T Stage, n (%) | | | | <0.001 |
| T1 | 0 | 0 | 0 |  |
| T1a | 61 (3.3) | 21 (4.8) | 30 (4.0) |  |
| T1b | 100 (5.4) | 39 (8.9) | 40 (5.3) |  |
| T1c | 11(0.6) | 2 (0.5) | 3 (0.4) |  |
| T2 | 0 | 0 | 0 |  |
| T2a | 382 (20.5) | 156 (35.8) | 125 (16.5) |  |
| T2b | 167 (8.9) | 42 (9.6) | 61 (8.1) |  |
| T3 | 557 (29.8) | 117 (26.8) | 245 (32.4) |  |
| T4 | 562 (30.1) | 59 (13.5) | 245 (32.4) |  |
| TX | 14 (0.8) | 0 | 6 (0.8) |  |
| N/A | 12 (0.6) | 0 | 2 (0.3) |  |
| N Stage, n (%) | | | | <0.001 |
| N0 | 134 (7.2) | 28 (6.4) | 42 (5.6) |  |
| N1 | 171 (9.2) | 60 (13.8) | 58 (7.7) |  |
| N2 | 1009 (54.1) | 331 (75.9) | 332 (43.9) |  |
| N3 | 529 (28.3) | 16 (3.7) | 320 (42.3) |  |
| NX | 23 (1.2) | 1 (0.2) | 5 (0.7) |  |
| EGFR testing, n (%) | 865 | 274 | 364 | <0.001 |
| Mutation | 297 (34.3) | 118 (43.1) | 105 (28.9) |  |
| No mutation | 513 (59.3) | 149 (54.4) | 250 (68.7) |  |
| Uncertain | 55 (6.4) | 7 (2.6) | 9 (2.5) |  |
| PD-L1 testing, n (%)^d^ | 292 | 93 | 143 | 0.551 |
| Negative | 145 (49.7) | 44 (47.3) | 62 (43.4) |  |
| Positive^d^ | 147 (50.3) | 49 (52.7) | 81 (56.6) |  |

AJCC=American Joint Committee on Cancer; BMI=Body mass index; ECOG=Eastern Cooperative Oncology Group; EGFR=Epidermal growth factor receptor; N=Number of patients; n=Number of patients in the subcategories; NSCLC=Non‑small cell lung cancer; PD‑L1=Programmed death-ligand 1.

^a^Current smoker: patients who smoke 1 or more tobacco products. Never-smoker: patients who have never smoked more than 20 grams of tobacco (1 pack of 20 cigarettes) in their lifetime. Ex-smoker: Patients who stopped smoking ≥365 days ago.

^b^The definition for “Resectable” and Unresectable” was based on the data in the electronic Case Record Form as follows:

Resectable: the response to “Did the patient undergo curative surgical resection” is yes.

Unresectable: the response to “Did the patient undergo curative surgical resection” is no and one of the following has to be ticked: “considered not correct management” or “medically unfit/comorbid reason” or other: “unresectable Stage III NSCLC

^c^others include (not exhaustive): adenosquamous carcinoma; lymphoepitheloid; squamous metaplasia with atypia, not otherwise specified; lymphoepithelioma-like carcinoma; carcinosarcoma; anaplastic carcinoma; sarcomatoid carcinoma; squamous cell carcinoma with sarcomatoid element; pleomorphic carcinoma; “carcinoma”; undifferentiated carcinoma; pleomorphic carcinoma; small cell carcinoma; epithelial epitheloid; (large cell) neuroendocrine carcinoma; adenoid cystic carcinoma; dual squamous and glandular differentiation.

^d^The investigators reported tests positive or negative according to their local pathology standards and procedures.

Note: Unknown and missing data are not included

**Supplementary Table S3.** Summary of EGFR mutations and PD-L1 analyses as per disease stage (AJCC 7^th^ Edition) and resection status in KINDLE-Asia

| **Biomarker testing** | **Stage IIIA** | | **Stage IIIB** | | **Total** | |
| --- | --- | --- | --- | --- | --- | --- |
|  | **Resectable** (N=379) | **Unresectable** (N=320) | **Resectable** (N=46) | **Unresectable** (N=417) | **Resectable** (N=425) | **Unresectable** (N=737) |
| EGFR testing, n (%) | 243 (64.1) | 129 (40.3) | 24 (52.2) | 226 (54.2) | 267 (62.8) | 355 (48.2) |
| Mutation present | 112 (46.1) | 39 (30.2) | 6 (25.0) | 65 (28.8) | 118 (44.2) | 104 (29.3) |
| No mutation | 124 (51.0) | 88 (68.2) | 18 (75.0) | 156 (69.0) | 142 (53.2) | 244 (68.7) |
| Uncertain | 7 (2.9) | 2 (1.6) | 0 | 5 (2.2) | 7 (2.6) | 7 (2.0) |
| Missing | 136 | 191 | 22 | 191 | 158 | 382 |
| PD-L1 testing^a^, n (%) | 85 (22.4) | 66 (20.6) | 7 (15.2) | 72 (17.3) | 92 (21.6) | 138 (18.7) |
| Negative | 40 (47.1) | 36 (54.5) | 3 (42.9) | 24 (33.3) | 43 (46.7) | 60 (43.5) |
| Positive^b^ | 45 (52.9) | 30 (45.5) | 4 (57.1) | 48 (66.7) | 49 (53.3) | 78 (56.5) |
| Missing | 294 | 254 | 39 | 345 | 333 | 599 |

AJCC=American Joint Committee on Cancer; EGFR=Epidermal growth factor receptor; N=Number of patients; n=Number of patients in the subcategories; PD-L1=Programmed death-ligand 1.

^a^ The investigators reported tests positive or negative according to their local pathology standards and procedures.

^b^ PD-L1 expression ≥1%.

Patients with data available for both resectability status and staging are included in this analysis.

**Supplementary Table S4.** Characteristics of patients with and without EGFR mutations in stage III NSCLC in KINDLE-Asia

| **Characteristics** | **EGFR mutations** (N=297) | **No** **EGFR mutations** (N=513) | **p‑value** |
| --- | --- | --- | --- |
| Age (years) |  |  |  |
| Number of patients^†^ | 296 | 512 | 0.1466 |
| Median (range) | 64.0 (25.0-90.0 ) | 63.0 (24.0-92.0) |  |
| Gender, n (%) |  |  | <0.0001 |
| Female | 153 (51.5) | 123 (24) |  |
| Male | 144 (48.5) | 390 (76) |  |
| Smoking Status, n (%) |  |  | <0.0001 |
| Current smoker | 34 (11.4) | 166 (32.4) |  |
| Ex-smoker | 55 (18.5) | 179 (34.9) |  |
| Never-smoker | 175 (58.9) | 130 (25.3) |  |
| Disease Stage (AJCC, 7th edition), n (%) |  |  | 0.0097 |
| Stage IIIA | 181 (62.2) | 261 (52.7) |  |
| Stage IIIB | 110 (37.8) | 234 (47.3) |  |
| Histology Type, n (%) |  |  | <0.0001 |
| Adenocarcinoma | 274 (92.3) | 362 (70.6) |  |
| Bronchiole-alveolar | 0 | 1 (0.2) |  |
| Epidermoid or Squamous Cell Carcinoma | 14 (4.7) | 106 (20.7) |  |
| Large Cell Carcinoma | 2 (0.7) | 7 (1.4) |  |
| Mixed | 3 (1.0) | 5 (1.0) |  |
| Other^a^, specify | 2 (0.7) | 25 (4.9) |  |
| ECOG Performance Status, n (%) |  |  | 0.0927 |
| 0 | 72 (24.2) | 91 (17.7) |  |
| 1 | 101 (34.0) | 186 (36.3) |  |
| 2 | 7 (2.4) | 24 (4.7) |  |
| 3 | 5 (1.7) | 5 (1.0) |  |
| 4 | 0 | 0 |  |
| Resectability, n (%)^b^ |  |  | 0.0002 |
| Resectable | 118 (52.9) | 149 (37.3) |  |
| Unresectable | 105 (47.1) | 250 (62.7) |  |

AJCC=American Joint Committee on Cancer; ECOG=Eastern Cooperative Oncology Group; EGFR=Epidermal growth factor receptor; N=Number of patients; n=Number of patients in the subcategories^;^ NSCLC=Non-small cell lung cancer.

^a^ “others” include (not exhaustive): adenosquamous carcinoma; lymphoepitheloid; squamous metaplasia with atypia, not otherwise specified; lymphoepithelioma-like carcinoma; carcinosarcoma; anaplastic carcinoma; sarcomatoid carcinoma; squamous cell carcinoma with sarcomatoid element; pleomorphic carcinoma; “carcinoma”; undifferentiated carcinoma; pleomorphic carcinoma; small cell carcinoma; epithelial epithelioid; (large cell) neuroendocrine carcinoma; adenoid cystic carcinoma; dual squamous and glandular differentiation.

^b^The definition for “Resectable” and Unresectable” was based on the data in the electronic Case Record Form as follows:

Resectable= the response to “Did the patient undergo curative surgical resection” is yes.

Unresectable= the response to “Did the patient undergo curative surgical resection” is no and one of the following has to be ticked: “considered not correct management” or “medically unfit/comorbid reason” or other: “unresectable Stage III NSCLC

Note: Unknown and missing data are not included.

†: One patient in the EGFR mutation group and the other patient in the EGFR wild type (without mutations) group do not have the original diagnosis date, hence they are excluded from the age analysis.

**Supplementary Table S5.** Treatment patterns as per disease stage (7^th^ Edition AJCC) in KINDLE-Asia

| **Treatment, n (%)** | **Stage IIIA** (N**=**976) | | | **Stage IIIB** (N=808) | | | **Overall**  (N=1874) | | |
| --- | --- | --- | --- | --- | --- | --- | --- | --- | --- |
|  | **Initial therapy** | **Second line** | **Third line** | **Initial therapy** | **Second line** | **Third line** | **Initial therapy** | **Second line** | **Third line** |
| **Curative surgery-based therapy** | | | | | | | | | |
| Surgery | 38 (4.1) | 6 (1.6) | 2 (1.4) | 2 (0.3) | 0 | 2 (1.5) | 41 (2.3) | 7 (0.9) | 4 (1.4) |
| cCRT+Surgery | 7 (0.8) | 0 | 0 | 4 (0.5) | 0 | 0 | 11 (0.6) | 0 | 0 |
| CT+Surgery | 18 (1.9) | 0 | 0 | 7 (0.9) | 2 (0.6) | 1 (0.7) | 25 (1.4) | 2 (0.3) | 1 (0.4) |
| Surgery+cCRT | 24 (2.6) | 0 | 0 | 2 (0.3) | 1 (0.3) | 0 | 26 (1.5) | 1 (0.1) | 0 |
| Surgery+sCRT | 67 (7.2) | 0 | 0 | 7 (0.9) | 1 (0.3) | 0 | 76 (4.3) | 1 (0.1) | 0 |
| Surgery+CT | 87 (9.3) | 1 (0.3) | 0 | 10 (1.3) | 1 (0.3) | 0 | 99 (5.6) | 2 (0.3) | 0 |
| Surgery+RT | 15 (1.6) | 3 (0.8) | 2 (1.4) | 2 (0.3) | 5 (1.5) | 0 | 17 (1.0) | 9 (1.2) | 2 (0.7) |
| Other surgery | 93 (10.0) | 5 (1.3) | 3 (2.1) | 19 (2.5) | 6 (1.8) | 1 (0.7) | 116 (6.5) | 11 (1.5) | 4 (1.4) |
| **cCRT-based therapy** | | | | | | | | | |
| cCRT | 247 (26.5) | 21 (5.5) | 5 (3.4) | 254 (33.2) | 15 (4.5) | 0 | 519 (29.3) | 36 (4.8) | 6 (2.1) |
| cCRT+CT | 17 (1.8) | 1 (0.3) | 2 (1.4) | 31 (4.0) | 3 (0.9) | 0 | 51 (2.9) | 4 (0.5) | 2 (0.7) |
| cCRT+RT | 8 (0.9) | 1 (0.3) | 0 | 3 (0.4) | 0 | 0 | 11 (0.6) | 1 (0.1) | 0 |
| cCRT+Targeted therapy | 2 (0.2) | 0 | 0 | 6 (0.8) | 1 (0.3) | 0 | 8 (0.5) | 1 (0.1) | 0 |
| cCRT+IO | 4 (0.4) | 2 (0.5) | 0 | 4 (0.5) | 0 | 0 | 8 (0.5) | 2 (0.3) | 0 |
| Other cCRT | 3 (0.3) | 1 (0.3) | 0 | 4 (0.5) | 1 (0.3) | 0 | 8 (0.5) | 2 (0.3) | 0 |
| **sCRT-based therapy** | | | | | | | | | |
| sCRT | 82 (8.8) | 19 (4.9) | 9 (6.2) | 78 (10.2) | 15 (4.5) | 13 (9.7) | 169 (9.5) | 34 (4.6) | 22 (7.8) |
| sCRT+CT | 1 (0.1) | 2 (0.5) | 2 (1.4) | 4 (0.5) | 2 (0.6) | 1 (0.7) | 5 (0.3) | 4 (0.5) | 3 (1.1) |
| sCRT+RT | 0 | 0 | 1 (0.7) | 2 (0.3) | 0 | 0 | 2 (0.1) | 0 | 1 (0.4) |
| sCRT+IO | 2 (0.2) | 3 (0.8) | 0 | 0 | 0 | 0 | 3 (0.2) | 4 (0.5) | 0 |
| sCRT+Targeted therapy | 1 (0.1) | 2 (0.5) | 1 (0.7) | 1 (0.1) | 2 (0.6) | 0 | 2 (0.1) | 4 (0.5) | 1 (0.4) |
| Other sCRT | 1 (0.1) | 0 | 0 | 3 (0.4) | 0 | 0 | 4 (0.2) | 0 | 0 |
| **Systemic therapy** | | | | | | | | | |
| CT | 100 (10.7) | 133 (34.5) | 47 (32.4) | 150 (19.6) | 137 (40.7) | 56 (41.8) | 262 (14.8) | 282 (37.8) | 104 (36.9) |
| CT+IO | 1 (0.1) | 6 (1.6) | 1 (0.7) | 2 (0.3) | 4 (1.2) | 2 (1.5) | 3 (0.2) | 10 (1.3) | 3 (1.1) |
| CT+Targeted therapy | 3 (0.3) | 9 (2.3) | 2 (1.4) | 8 (1.0) | 9 (2.7) | 4 (3.0) | 12 (0.7) | 18 (2.4) | 6 (2.1) |
| CT+  Targeted therapy+IO | 0 | 0 | 1 (0.7) | 1 (0.1) | 0 | 0 | 1 (<0.1) | 0 | 1 (0.4) |
| IO | 1 (0.1) | 15 (3.9) | 8 (5.5) | 3 (0.4) | 19 (5.6) | 7 (5.2) | 4 (0.2) | 35 (4.7) | 15 (5.3) |
| Targeted therapy | 22 (2.4) | 57 (14.8) | 18 (12.4) | 58 (7.6) | 41 (12.2) | 13 (9.7) | 81 (4.6) | 100 (13.4) | 31 (11.0) |
| Targeted therapy+IO | 0 | 0 | 0 | 0 | 0 | 1 (0.7) | 0 | 0 | 1 (0.4) |
| **RT-based therapy** | | | | | | | | | |
| RT | 70 (7.5) | 77 (20.0) | 29 (20.0) | 63 (8.2) | 58 (17.2) | 29 (21.6) | 151 (8.5) | 141 (18.9) | 59 (20.9) |
| RT+IO | 5 (0.5) | 5 (1.3) | 1 (0.7) | 10 (1.3) | 3 (0.9) | 1 (0.7) | 15 (0.8) | 8 (1.1) | 2 (0.7) |
| RT+Targeted therapy | 11 (1.2) | 15 (3.9) | 10 (6.9) | 23 (3.0) | 11 (3.3) | 3 (2.2) | 35 (2.0) | 26 (3.5) | 13 (4.6) |
| RT+Targeted therapy+IO | 1 (0.1) | 1 (0.3) | 1 (0.7) | 5 (0.7) | 0 | 0 | 6 (0.3) | 1 (0.1) | 1 (0.4) |
| Total | 931 (95.4) | 385 (39.5) | 145 (14.9) | 766 (94.8) | 337 (41.7) | 134 (16.6) | 1771 (94.5) | 746 (39.8) | 282 (15.1) |

AJCC=American Joint Committee on Cancer; cCRT=Concurrent chemoradiotherapy; CT=Chemotherapy; IO=Immuno-oncology; N=Number of patients; n=Number of patients in the subcategories; RT=Radiotherapy; sCRT=Sequential chemoradiotherapy.

The treatment pattern definitions are based on the available patterns from the full analysis set for first line used until 1^st^ progressive disease.

Surgery: only surgery was used, cCRT+Surgery: cCRT and surgery were used in sequence, CT+Surgery: chemotherapy and surgery were used in sequence, Surgery+sCRT: surgery and sCRT were used in sequence, Surgery+CT: surgery and chemotherapy were used in sequence, Surgery+RT: surgery and radiotherapy were used in sequence, Other Surgery: other therapies used in combination with surgery, cCRT: only cCRT was used, cCRT+CT: cCRT and chemotherapy were used, cCRT+RT: cCRT and radiotherapy were used, cCRT+targeted therapy: cCRT and targeted therapy were used, cCRT+IO: cCRT and immunotherapy were used, Other cCRT: other therapies excluding surgery used in combination with cCRT, sCRT: only sCRT was used, sCRT+CT: sCRT and chemotherapy were used, sCRT+RT: sCRT and radiotherapy were used, sCRT+IO: sCRT and immunotherapy were used, sCRT+targeted therapy:sCRT and targeted therapy were used, Other sCRT: other therapies excluding surgery used in combination with cCRT, CT: only chemotherapy was used, CT+IO: chemotherapy and immunotherapy were used, CT+targeted therapy: chemotherapy and targeted therapy were used, CT+targeted therapy+IO: chemotherapy, targeted therapy and immunotherapy were used, IO: only immunotherapy was used, RT: only radiotherapy was used, RT+IO: radiotherapy and immunotherapy were used, RT+targeted therapy: radiotherapy and targeted therapy were used, RT+targeted therapy+IO: radiotherapy, targeted therapy and immunotherapy were used, Targeted therapy: only targeted therapy was used.

**Supplementary Table S6.** Treatment patterns according to resection status in KINDLE-Asia

| **Treatment, n (%)** | **Resectable** (N=437) | | | **Unresectable** (N=758) | | |
| --- | --- | --- | --- | --- | --- | --- |
|  | **Initial therapy** | **Second line** | **Third line** | **Initial therapy** | **Second line** | **Third line** |
| Surgery | 33 (7.9) | 7 (4.1) | 2 (2.6) | 1 (0.1) | 0 | 1 (0.6) |
| Surgery+cCRT | 25 (6.0) | 0 | 0 | 1 (0.1) | 1 (0.3) | 0 |
| Surgery+sCRT | 67 (16.0) | 0 | 0 | 0 | 1 (0.3) | 0 |
| Surgery+CT | 84 (20.0) | 2 (1.2) | 0 | 3 (0.4) | 0 | 0 |
| Surgery+RT | 16 (3.8) | 4 (2.4) | 1 (1.3) | 0 | 3 (0.9) | 1 (0.6) |
| cCRT+Surgery | 9 (2.1) | 0 | 0 | 1 (0.1) | 0 | 0 |
| CT+Surgery | 12 (2.9) | 0 | 0 | 3 (0.4) | 1 (0.3) | 1 (0.6) |
| Other surgery | 93 (22.2) | 5 (2.9) | 2 (2.6) | 9 (1.2) | 4 (1.2) | 2 (1.3) |
| cCRT | 10 (2.4) | 12 (7.1) | 3 (3.9) | 323 (44.7) | 17 (5.0) | 1 (0.6) |
| cCRT+CT | 3 (0.7) | 0 | 2 (2.6) | 25 (3.5) | 4 (1.2) | 0 |
| cCRT+RT | 3 (0.7) | 1 (0.6) | 0 | 5 (0.7) | 0 | 0 |
| cCRT+IO | 0 | 2 (1.2) | 0 | 4 (0.6) | 0 | 0 |
| cCRT+Targeted therapy | 0 | 0 | 0 | 6 (0.8) | 0 | 0 |
| Other cCRT | 0 | 0 | 0 | 7 (1.0) | 2 (0.6) | 0 |
| sCRT | 20 (4.8) | 8 (4.7) | 2 (2.6) | 64 (8.9) | 14 (4.1) | 13 (8.4) |
| sCRT+CT | 0 | 2 (1.2) | 0 | 2 (0.3) | 1 (0.3) | 3 (1.9) |
| sCRT+RT | 0 | 0 | 0 | 1 (0.1) | 0 | 1 (0.6) |
| sCRT+IO | 0 | 0 | 0 | 1 (0.1) | 3 (0.9) | 0 |
| sCRT+Targeted therapy | 0 | 0 | 1 (1.3) | 1 (0.1) | 2 (0.6) | 0 |
| Other sCRT | 0 | 0 | 0 | 2 (0.3) | 0 | 0 |
| CT | 37 (8.8) | 32 (18.8) | 23 (29.9) | 110 (15.2) | 141 (41.2) | 56 (36.4) |
| CT+IO | 1 (0.2) | 1 (0.6) | 1 (1.3) | 2 (0.3) | 8 (2.3) | 1 (0.6) |
| CT+  Targeted therapy | 1 (0.2) | 4 (2.4) | 1 (1.3) | 5 (0.7) | 5 (1.5) | 4 (2.6) |
| CT+Targeted therapy+IO | 0 | 0 | 1 (1.3) | 1 (0.1) | 0 | 0 |
| RT | 3 (0.7) | 33 (19.4) | 18 (23.4) | 85 (11.8) | 69 (20.2) | 34 (22.1) |
| RT+IO | 0 | 2 (1.2) | 1 (1.3) | 5 (0.7) | 2 (0.6) | 0 |
| RT+Targeted therapy | 1 (0.2) | 11 (6.5) | 6 (7.8) | 13 (1.8) | 7 (2.0) | 6 (3.9) |
| RT+Targeted therapy+IO | 0 | 1 (0.6) | 1 (1.3) | 2 (0.3) | 0 | 0 |
| IO | 0 | 3 (1.8) | 2 (2.6) | 1 (0.1) | 18 (5.3) | 11 (7.1) |
| Targeted therapy | 1 (0.2) | 40 (23.5) | 10 (13.0) | 40 (5.5) | 39 (11.4) | 18 (11.7) |
| Targeted therapy+IO | 0 | 0 | 0 | 0 | 0 | 1 (0.6) |
| Total | 419 (95.9) | 170 (38.9) | 77 (17.6) | 723 (95.4) | 342 (45.1) | 154 (20.3) |

cCRT=Concurrent chemoradiotherapy; CT=Chemotherapy; IO=Immuno-oncology; N=Number of patients; n=Number of patients in the subcategories; RT=Radiotherapy; sCRT=Sequential chemoradiotherapy.

The treatment pattern definitions are based on the available patterns from the full analysis set for first line used until 1st progressive disease.

Surgery: only surgery was used, cCRT+Surgery: cCRT and surgery were used in sequence, CT+surgery: chemotherapy and surgery were used in sequence, Surgery+sCRT: surgery and sCRT were used in sequence, Surgery+CT: surgery and chemotherapy were used in sequence, Surgery+RT: surgery and radiotherapy were used in sequence, Other Surgery: other therapies used in combination with surgery, cCRT: only cCRT was used, cCRT+CT: cCRT and chemotherapy were used, cCRT+RT: cCRT and radiotherapy were used, cCRT+targeted therapy: cCRT and targeted therapy were used, cCRT+IO: cCRT and immunotherapy were used, Other cCRT: other therapies excluding surgery used in combination with cCRT, sCRT: only sCRT was used, sCRT+CT: sCRT and chemotherapy were used, sCRT+RT: sCRT and radiotherapy were used, sCRT+IO: sCRT and immunotherapy were used, sCRT+targeted therapy: sCRT and targeted therapy were used, Other sCRT: other therapies excluding surgery used in combination with cCRT, CT: only chemotherapy was used, CT+IO: chemotherapy and immunotherapy were used, CT+targeted therapy: chemotherapy and targeted therapy were used, CT+targeted therapy+IO: chemotherapy, targeted therapy and immunotherapy were used, IO: only immunotherapy was used, RT: only radiotherapy was used, RT+IO: radiotherapy and immunotherapy were used, RT+targeted therapy: radiotherapy and targeted therapy were used, RT+targeted therapy+IO: radiotherapy, targeted therapy and immunotherapy were used, targeted therapy: only targeted therapy was used.

**Supplementary Table S7.** Characteristics of patients with unresectable stage III NSCLC receiving initial treatment with cCRT or Targeted therapy in KINDLE-Asia

| **Characteristics** | **cCRT (initial therapy)**  (N=323) | **Targeted treatment (initial therapy)** (N=40) | **p‑value** |
| --- | --- | --- | --- |
| Age (Years) |  |  | <0.001 |
| Number of patients | 323 | 40 |  |
| Median (range) | 63.7 (32.0-84.0) | 72.1 (43.0-90.0) |  |
| Gender n (%) |  |  | 0.0001 |
| Female | 70 (21.7) | 20 (50) |  |
| Male | 253 (78.3) | 20 (50) |  |
| Tobacco Smoking n (%) |  |  | <0.001 |
| Current smoker | 116 (35.9) | 4 (10) |  |
| Ex-smoker | 121 (37.5) | 7 (17.5) |  |
| Never-smoker | 79 (24.5) | 27 (67.5) |  |
| Disease stage (AJCC 7th edition) n (%) |  |  | 0.008 |
| Stage IIIA | 153 (48.1) | 8 (20.5) |  |
| Stage IIIB | 165 (51.9) | 31 (79.5) |  |
| Histology Type n (%) |  |  | 0.002 |
| Adenocarcinoma | 162 (50.2) | 38 (95) |  |
| Bronchiole-alveolar | 0 | 0 |  |
| Epidermoid or Squamous Cell Carcinoma | 116 (35.9) | 2 (5) |  |
| Large Cell Carcinoma | 5 (1.5) | 0 |  |
| Mixed | 4 (1.2) | 0 |  |
| Other^a^, specify | 24 (6.6) | 24 (7.4) |  |
| ECOG Performance Status n (%) |  |  | <0.001 |
| 0 | 64 (27.9) | 3 (12.5) |  |
| 1 | 147 (64.2) | 12 (50) |  |
| 2 | 17 (7.4) | 4 (16.7) |  |
| 3 | 1 (0.4) | 5 (20.8) |  |
| 4 | 0 | 0 |  |
| Resectability n (%) |  |  |  |
| Resectable | 0 | 0 |  |
| Unresectable | 323 (100) | 40 (100) |  |

AJCC=American Joint Committee on Cancer; cCRT=Concurrent chemoradiotherapy; ECOG=Eastern Cooperative Oncology Group; N=Number of patients; n=Number of patients in the subcategories^;^ NSCLC=Non-small cell lung cancer.

^a^ “others” include (not exhaustive): adenosquamous carcinoma; lymphoepitheloid; squamous metaplasia with atypia, not otherwise specified; lymphoepithelioma-like carcinoma; carcinosarcoma; anaplastic carcinoma; sarcomatoid carcinoma; squamous cell carcinoma with sarcomatoid element; pleomorphic carcinoma; “carcinoma”; undifferentiated carcinoma; pleomorphic carcinoma; small cell carcinoma; epithelial epithelioid; (large cell) neuroendocrine carcinoma; adenoid cystic carcinoma; dual squamous and glandular differentiation. Note: Unknown and missing data are not included.

**Supplementary Table S8.**Survival outcomes with initial treatment according to resection status in KINDLE-Asia

|  | **Resectable** (N=437) | | | | **Unresectable** (N=758) | | | |
| --- | --- | --- | --- | --- | --- | --- | --- | --- |
| **Treatment** | **N** | **mPFS months (95% CI)** | **N** | **mOS months (95% CI)** | **N** | **mPFS months (95% CI]** | **N** | **mOS months (95% CI)** |
| Surgery | 33 | 15.4 (11.24-24.41) | 33 | 32.1 (23.26-66.73) | 1 | 7.1 (NC-NC) | 1 | 11.0 (NC-NC) |
| Surgery+cCRT | 25 | 19.9 (10.64-35.81) | 25 | 41.9 (30.39-NC) | 1 | 3.5 (NC-NC) | 1 | 3.5 (NC-NC) |
| Surgery+sCRT | 67 | 29.3 (18.00-NC) | 67 | NC (43.83-NC) | 0 | - | 0 | - |
| Surgery+CT | 84 | 17.8 (12.06-25.03) | 84 | 57.9 (42.94-NC) | 3 | 8.9 (8.51-18.76) | 3 | 21.0 (18.86-NC) |
| Other Surgery | 93 | 29.9 (21.13-43.20) | 93 | NC (NC-NC) | 9 | 25.6 (7.82-NC) | 9 | 40.4 (17.58-NC) |
| cCRT | 10 | 12.8 (5.09-19.12) | 10 | 25.8 (16.33-NC) | 323 | 11.3 (9.40-13.04) | 323 | 39.2 (32.36-50.79) |
| cCRT+CT | 3 | 10.9 (4.21-12.35) | 3 | 14.6 (14.55-35.58) | 25 | 10.1 (7.79-15.11) | 25 | 32.8 (18.17-NC) |
| sCRT | 20 | 12.6 (8.18-19.06) | 20 | NC (18.30-NC) | 64 | 12.5 (9.43-14.95) | 64 | 26.6 (18.56-36.70) |
| CT | 37 | 15.1 (6.74-23.72) | 37 | 65.4 (43.83-NC) | 110 | 6.7 (5.91-8.71) | 110 | 25.1 (17.31-42.61) |
| RT | 3 | 8.9 (1.61-12.85) | 3 | NC (8.87-NC) | 85 | 10.4 (7.39-12.19) | 85 | 16.8 (12.19-27.24) |
| Targeted therapy | 1 | 2.8 (NC-NC) | 1 | NC (NC-NC) | 40 | 13.8 (6.44-16.56) | 40 | 24.0 (14.62-30.52) |

cCRT=Concurrent chemoradiotherapy; CI=Confidence interval; CT=Chemotherapy; IO=immune-oncology; mOS=Median overall survival; mPFS=Median progression-free survival; N=Number of patients; NC=Not calculable; RT=Radiotherapy; sCRT=Sequential chemoradiotherapy; ‘-'=not available.

The treatment pattern definitions are based on the available patterns from the full analysis set for first line used until 1^st^ progressive disease

Data is not shown if ‘N’ is <20 in both resectable and unresectable groups

Surgery: only surgery was used, Surgery+sCRT: surgery and sCRT were used in sequence, Surgery+cCRT: surgery and cCRT were used in sequence, Other Surgery: other therapies used in combination with surgery, cCRT: only cCRT was used, cCRT+CT: cCRT and chemotherapy were used, sCRT: only sCRT was used, CT: only chemotherapy was used, RT: only radiotherapy was used, Targeted therapy: only targeted therapy was used.

**Supplementary Table S9.** Survival outcomes with different initial treatment patterns in unresectable stage III NSCLC

|  | **mPFS months (95% CI)** | | | **mOS months (95% CI)** | | |
| --- | --- | --- | --- | --- | --- | --- |
| **Group1 vs Group2** | **Group1** | **Group2** | **p-value (log-rank)** | **Group1** | **Group2** | **p-value (log‑rank)** |
| cCRT vs CT | 11.3 (9.40-13.04) | 6.7 (5.91-8.71) | **<0.0001** | 39.2 (32.36-50.79) | 25.1 (17.31-42.61) | **0.0171** |
| cCRT vs sCRT | 11.3 (9.40-13.04) | 12.5 (9.43-14.95) | 0.6324 | 39.2 (32.36-50.79) | 26.6 (18.56-36.70) | **0.0384** |
| cCRT vs RT | 11.3 (9.40-13.04) | 10.4 (7.39-12.19) | 0.0485 | 39.2 (32.36-50.79) | 16.8 (12.19-27.24) | **<0.0001** |
| cCRT vs Surgery+CT | 11.3 (9.40-13.04) | 8.9 (8.51-18.76) | 0.5496 | 39.2 (32.36-50.79) | 21.0 (18.86-NC) | 0.3892 |
| cCRT vs Targeted therapy | 11.3 (9.40-13.04) | 13.8 (6.44-16.56) | 0.2068 | 39.2 (32.36-50.79) | 24.0 (14.62-30.52) | **0.0006** |
| CT vs sCRT | 6.7 (5.91-8.71) | 12.5 (9.43-14.95) | **0.0078** | 25.1 (17.31-42.61) | 26.6 (18.56-36.70) | 0.9617 |
| CT vs RT | 6.7 (5.91-8.71) | 10.4 (7.39-12.19) | 0.1339 | 25.1 (17.31-42.61) | 16.8 (12.19-27.24) | 0.0511 |
| CT vs Surgery+CT | 6.7 (5.91-8.71) | 8.9 (8.51-18.76) | 0.7287 | 25.1 (17.31-42.61) | 21.0 (18.86-NC) | 0.8684 |
| CT vs Targeted therapy | 6.7 (5.91-8.71) | 13.8 (6.44-16.56) | 0.1033 | 25.1 (17.31-42.61) | 24.0 (14.62-30.52) | 0.2184 |
| sCRT vs RT | 12.5 (9.43-14.95) | 10.4 (7.39-12.19) | 0.2692 | 26.6 (18.56-36.70) | 16.8 (12.19-27.24) | 0.1073 |
| sCRT vs Surgery+CT | 12.5 (9.43-14.95) | 8.9 (8.51-18.76) | 0.6737 | 26.6 (18.56-36.70) | 21.0 (18.86-NC) | 0.6902 |
| sCRT vs Targeted therapy | 12.5 (9.43-14.95) | 13.8 (6.44-16.56) | 0.7182 | 26.6 (18.56-36.70) | 24.0 (14.62-30.52) | 0.1933 |
| RT vs Surgery+CT | 10.4 (7.39-12.19) | 8.9 (8.51-18.76) | 0.8476 | 16.8 (12.19-27.24) | 21.0 (18.86-NC) | 0.7591 |
| RT vs Targeted therapy | 10.4 (7.39-12.19) | 13.8 (6.44-16.56) | 0.6469 | 16.8 (12.19-27.24) | 24.0 (14.62-30.52) | 0.8220 |
| Surgery+CT vs Targeted therapy | 8.9 (8.51-18.76) | 13.8 (6.44-16.56) | 0.6087 | 21.0 (18.86-NC) | 24.0 (14.62-30.52) | 0.9680 |

cCRT=Concurrent chemoradiotherapy; CI=Confidence interval; CT=Chemotherapy; mOS=Median overall survival; mPFS=Median progression-free survival; NC=Not calculable; NSCLC=Non‑small cell lung cancer, RT=Radiotherapy sCRT=Sequential chemoradiotherapy; vs= Versus

Values in bold indicate significant difference (p<0.05).

**Supplementary Table S10.** Survival outcomes with initial treatment according to disease stage (AJCC 7^th^ Edition) in KINDLE-Asia

|  | **Stage IIIA** (N=976) | | | | **Stage IIIB** (N=808) | | | |
| --- | --- | --- | --- | --- | --- | --- | --- | --- |
| **Treatment** | **N** | **mPFS months (95% CI)** | **N** | **mOS months (95% CI)** | **N** | **mPFS months (95% CI)** | **N** | **mOS months (95% CI)** |
| Surgery | 38 | 15.8 (12.71-28.12) | 38 | 37.1 (23.26-66.73) | 2 | 12.8 (1.18- 24.41) | 2 | 12.8 (1.18-24.41) |
| Surgery+cCRT | 24 | 19.4 (10.35-35.81) | 24 | 41.3 (25.95-NC) | 2 | 17.7 (10.61-24.71) | 2 | NC (NC-NC) |
| Surgery+sCRT | 67 | 29.3 (19.61-NC) | 67 | NC (NC-NC) | 7 | NC (11.17-NC) | 7 | NC (29.70-NC) |
| Surgery+CT | 87 | 15.6 (11.66-21.91) | 87 | 57.9 (37.82-NC) | 10 | 20.7 (1.48-NC) | 10 | NC (2.86-NC) |
| Surgery+RT | 14 | 27.8 (5.72-51.98) | 14 | 58.6 (14.49-NC) | 2 | NC (NC-NC) | 2 | NC (NC-NC) |
| cCRT+Surgery | 7 | 18.0 (7.26-NC) | 7 | NC (7.26-NC) | 4 | 14.7 (3.75-NC) | 4 | NC (4.11-NC) |
| CT+Surgery | 18 | 60.4 (17.15-NC) | 18 | NC (27.66-NC) | 7 | NC (7.13-NC) | 7 | NC (7.66-NC) |
| Other Surgery | 93 | 26.7 (20.17-39.95) | 93 | NC (45.01-NC) | 19 | 41.8 (18.20-NC) | 19 | NC (28.45-NC) |
| cCRT | 247 | 14.4 (12.45-18.04) | 247 | 50.8 (37.09-NC) | 254 | 9.3 (8.21-11.20) | 254 | 36.0 (28.62-47.38) |
| cCRT+CT | 17 | 11.2 (6.47-23.10) | 17 | 30.6 (18.79-NC) | 31 | 11.0 (9.13-15.70) | 31 | 32.8 (18.86-NC) |
| cCRT+RT | 8 | 9.2 (4.67-39.33) | 8 | 17.4 (9.20-NC) | 3 | 18.5 (1.41-NC) | 3 | NC (NC-NC) |
| cCRT+IO | 4 | 12.9 (8.08-NC) | 4 | 37.5 (13.44-37.52) | 4 | 14.1 (13.40-NC) | 4 | NC (NC-NC) |
| cCRT+Targeted therapy | 2 | 3.8 (1.51-6.18) | 2 | NC (NC-NC) | 6 | 10.1 (6.47-31.80) | 6 | 45.1 (11.96-NC) |
| Other cCRT | 3 | 18.3 (6.08-36.24) | 2 | NC (NC-NC) | 4 | 9.7 (4.93-11.07) | 4 | 11.3 (4.93-NC) |
| sCRT | 82 | 13.4 (10.74-14.95) | 82 | 29.0 (26.05-NC) | 78 | 9.4 (8.51-12.42) | 78 | 25.7 (17.18-NC) |
| sCRT+CT | 1 | 36.9 (NC-NC) | 1 | NC (NC-NC) | 4 | 17.9 (6.18-26.22) | 4 | 40.3 (6.51-42.61) |
| sCRT+RT | 0 | - | 0 | - | 2 | 13.8 (4.86-22.67) | 2 | 29.5 (4.86-54.08) |
| sCRT+IO | 2 | 9.1 (4.86-13.40) | 2 | NC (10.09-NC) | 0 | - | 0 | - |
| sCRT+Targeted therapy | 1 | NC (NC-NC) | 1 | NC (NC-NC) | 1 | 36.6 (NC-NC) | 1 | 55.7 (NC-NC) |
| Other sCRT | 1 | 36.9 (NC-NC) | 1 | NC (NC-NC) | 3 | 46.6 (8.87-46.62) | 3 | NC (17.48-NC) |
| CT | 100 | 9.6 (6.64-12.48) | 100 | 40.7 (29.24- 65.38) | 150 | 7.4 (6.51-9.30) | 149 | 24.2 (19.98-38.08) |
| CT+IO | 1 | NC (NC-NC) | 1 | NC (NC-NC) | 2 | 10.9 (8.31-13.40) | 2 | NC (26.41-NC) |
| CT+Targeted therapy | 3 | 14.7 (12.55-33.58) | 3 | NC (34.37-NC) | 8 | 18.4 (1.87-23.59) | 8 | NC (17.08-NC) |
| CT+Targeted therapy+IO | 0 | - | 0 | - | 1 | NC (NC-NC) | 1 | NC (NC-NC) |
| RT | 70 | 10.4 (8.38-12.52) | 70 | 30.3 (17.64-48.99) | 63 | 8.0 (4.60-10.84) | 63 | 13.0 (9.13-28.71) |
| RT+IO | 5 | 13.8 (10.15-38.64) | 5 | NC (10.15-NC) | 10 | NC (7.39-NC) | 10 | 35.4 (22.44-35.45) |
| RT+Targeted therapy | 11 | 15.2 (7.29-44.88) | 11 | 48.3 (9.36-NC) | 23 | 19.6 (6.54-23.49) | 22 | 36.8 (6.24-NC) |
| RT+Targeted therapy+IO | 1 | 13.0 (NC-NC) | 1 | NC (NC-NC) | 5 | 24.3 (11.17-NC) | 5 | NC (24.31-NC) |
| IO | 1 | NC (NC-NC) | 1 | NC (NC-NC) | 3 | 2.2 (1.87-22.34) | 3 | NC (13.17-NC) |
| Targeted Therapy | 22 | 10.8 (3.65-14.75) | 22 | 21.6 (14.62-30.52) | 58 | 10.5 (6.05-15.31) | 58 | 27.7 (24.18-50.33) |

AJCC=American Joint Committee on Cancer; cCRT=Concurrent chemoradiotherapy; CI=Confidence interval; CT=Chemotherapy; IO=Immuno-oncology; mOS=Median overall survival; mPFS=Median progression-free survival; N=Number of patients; NC=Not calculable; RT=Radiotherapy; sCRT=Sequential chemoradiotherapy; ‘-‘=Not available.

The treatment pattern definitions are based on the available patterns from the full analysis set for first line used until 1st progressive disease.

Surgery: only surgery was used, cCRT+Surgery: cCRT and surgery were used in sequence, CT+Surgery: chemotherapy and surgery were used in sequence, Surgery+sCRT: surgery and sCRT were used in sequence, Surgery+CT: surgery and chemotherapy were used in sequence, Surgery+RT: surgery and radiotherapy were used in sequence, Other Surgery: other therapies used in combination with surgery, cCRT: only cCRT was used, cCRT+CT: cCRT and chemotherapy were used, cCRT+RT: cCRT and radiotherapy were used, cCRT+Targeted therapy: cCRT and Targeted therapy were used, cCRT+IO: cCRT and immunotherapy were used, Other cCRT: other therapies excluding surgery used in combination with cCRT, sCRT: only sCRT was used, sCRT+CT: sCRT and chemotherapy were used, sCRT+RT: sCRT and radiotherapy were used, sCRT+IO: sCRT and immunotherapy were used, sCRT+Targeted therapy: sCRT and Targeted therapy were used, Other sCRT: other therapies excluding surgery used in combination with cCRT, CT: only chemotherapy was used, CT+IO: chemotherapy and immunotherapy were used, CT+Targeted therapy: chemotherapy and Targeted therapy were used, CT+Targeted therapy+IO: chemotherapy, Targeted therapy and immunotherapy were used, IO: only immunotherapy was used, RT: only radiotherapy was used, RT+IO: radiotherapy and immunotherapy were used, RT+Targeted therapy: radiotherapy and Targeted therapy were used, RT+Targeted therapy+IO: radiotherapy, Targeted therapy and immunotherapy were used, Targeted therapy: only Targeted therapy was used.

**Supplementary Table S11.** Summary of survival outcomes with initial treatment by EGFR mutation and resection status in KINDLE-Asia

| **Mutation status** | **Resectable** (N=267) | | | **Unresectable** (N=355) | | |
| --- | --- | --- | --- | --- | --- | --- |
|  | **N (%)** | **mPFS months (95% CI)** | **mOS months (95% CI)** | **N (%)** | **mPFS months (95% CI)** | **mOS months (95% CI)** |
| EGFR mutation | 118 (44.2) | 19.1 (15.7-24.1) | 59.5 (47.0-NA) | 105 (29.6) | 13.2 (10.6 -15.9) | 48.2 (37.3-NA) |
| No EGFR mutation | 149 (55.8) | 20.17 (16.40-28.5) | NA (44.5-NA) | 250 (70.4) | 9.57 (8.57-11.6) | 32.5 (25.8-43.5) |

CI=Confidence interval; EGFR=Epidermal growth factor receptor; mOS=Median overall survival; mPFS=Median progression-free survival; N=Number of patients; NA=Not applicable.

**Supplementary Table S12.** Univariate and multivariate analyses for progression-free survival in stage IIIA and IIIB (AJCC 7^th^ Edition) NSCLC patients in KINDLE-Asia

|  |  | **Stage IIIA** | | |  |  | **Stage IIIB** | | |  |
| --- | --- | --- | --- | --- | --- | --- | --- | --- | --- | --- |
|  | **N** | | **HR (95% CI)** | **p‑value** | | **N** | | **HR (95% CI)** | **p‑value** | |
| **Univariate Analyses** |  | |  |  | |  | |  |  | |
| Age>65 vs ≤65 years | 392 vs 538 | | 1.203 (1.029 to 1.405) | **0.0202** | | 298 vs 470 | | 1.164 (0.988 to 1.371) | 0.0686 | |
| ECOG ≤1 vs 2/3/4 | 512 vs 54 | | 0.588 (0.430 to 0.803) | **0.0009** | | 436 vs 59 | | 0.772 (0.574 to 1.039) | 0.0878 | |
| EGFRm vs EGFRwt | 172 vs 251 | | 1.040 (0.832 to 1.299) | 0.7314 | | 106 vs 221 | | 1.003 (0.781 to 1.288) | 0.9825 | |
| Male vs Female | 677 vs 253 | | 0.990 (0.834 to 1.175) | 0.9053 | | 581 vs 187 | | 1.045 (0.867 to 1.259) | 0.6467 | |
| Smoking history yes vs no | 586 vs 286 | | 1.071 (0.904 to 1.270) | 0.4263 | | 472 vs 203 | | 1.121 (0.931 to 1.350) | 0.2270 | |
| Resection yes vs no | 363 vs 305 | | 0.693 (0.578 to 0.832) | **<0.0001** | | 45 vs 399 | | 0.409 (0.270 to 0.619) | **<0.0001** | |
| Adenocarcinoma vs others | 535 vs 394 | | 0.940 (0.804 to 1.100) | 0.4410 | | 416 vs 350 | | 1.059 (0.901 to 1.244) | 0.4852 | |
| Surgery as a part of initial therapy yes vs no | 348 vs 582 | | 0.596 (0.505 to 0.702) | **<0.0001** | | 53 vs 715 | | 0.408 (0.277 to 0.601) | **<0.0001** | |
| cCRT as initial therapy yes vs no | 247 vs 683 | | 0.982 (0.822 to 1.172) | 0.8378 | | 254 vs 514 | | 0.940 (0.792 to 1.117) | 0.4826 | |
| CRT as initial therapy | 247 vs 82 | | 0.799 (0.596 to 1.072) | 0.1347 | | 254 vs 78 | | 0.963 (0.725 to 1.278) | 0.7920 | |
| Trimodality as a part of initial  therapy yes vs no | 120 vs 810 | | 0.630 (0.492 to 0.806) | **0.0002** | | 19 vs 749 | | 0.488 (0.261 to 0.911) | **0.0244** | |
| **Multivariate Analyses** |  | |  |  | |  | |  |  | |
| Age>65 vs ≤65 | 226 vs 312 | | 1.062 (0.861 to 1.310) | 0.5732 | | 187 vs 271 | | 1.119 (0.907 to 1.381) | 0.2924 | |
| ECOG ≤1 vs 2/3/4 | 489 vs 49 | | 0.681 (0.488 to 0.950) | **0.0237** | | 408 vs 50 | | 0.843 (0.614 to 1.159) | 0.2926 | |
| Male vs Female | 396 vs 142 | | 0.896 (0.646 to 1.244) | 0.5132 | | 349 vs 109 | | 1.022 (0.713 to 1.464) | 0.9067 | |
| Smoking history yes vs no | 362 vs 176 | | 1.246 (0.912 to 1.702) | 0.1663 | | 323 vs 135 | | 1.358 (0.971 to 1.900) | 0.0740 | |
| Adenocarcinoma vs others | 302 vs 236 | | 1.066 (0.846 to 1.343) | 0.5876 | | 252 vs 206 | | 1.179 (0.950 to 1.463) | 0.1346 | |
| Surgery as a part of initial therapy yes vs no | 191 vs 347 | | 0.513 (0.385 to 0.684) | **<0.0001** | | 26 vs 432 | | 0.303 (0.155 to 0.592) | **0.0005** | |
| cCRT as initial therapy yes vs no | 156 vs 382 | | 0.633 (0.491 to 0.816) | **0.0004** | | 179 vs 279 | | 0.838 (0.676 to 1.038) | 0.1061 | |
| Trimodality as a part of initial therapy yes vs no | 78 vs 460 | | 0.766 (0.521 to 1.124) | 0.1729 | | 7 vs 451 | | 3.147 (1.109 to 8.932) | **0.0312** | |

AJCC=American Joint Committee on Cancer; cCRT=Concurrent chemoradiotherapy; CI=Confidence interval; CRT=Chemoradiotherapy; ECOG=Eastern Cooperative Oncology Group; EGFRm=Epidermal growth factor receptor mutation; EGFRwt=Epidermal growth factor receptor wild‑type mutation; HR=Hazard ratio; N=Number of patients ^,^ vs=Versus

NSCLC=Non-small cell lung cancer.

Values in bold indicate significant difference (p<0.05).

**Supplementary Table S13.** Univariate and multivariate analyses for overall survival in stage IIIA and IIIB (AJCC 7^th^ Edition) NSCLC patients in KINDLE-Asia

|  |  | **Stage IIIA** | | |  |  | **Stage IIIB** | |  |
| --- | --- | --- | --- | --- | --- | --- | --- | --- | --- |
|  | **N** | | **HR (95% CI)** | **p‑value** | | **N** | | **HR (95% CI)** | **p‑value** |
| **Univariate Analyses** |  | |  |  | |  | |  |  |
| Age>65 vs ≤65 years | 392 vs 537 | | 1.487 (1.198 to 1.846) | **0.0003** | | 298 vs 468 | | 1.274 (1.024 to 1.584) | **0.0295** |
| ECOG ≤1 vs 2/3/4 | 511 vs 54 | | 0.483 (0.321 to 0.726) | **0.0005** | | 434 vs 59 | | 0.560 (0.389 to 0.806) | **0.0018** |
| EGFRm vs EGFRwt | 172 vs 251 | | 0.718 (0.516 to 1.000) | **0.0499** | | 105 vs 220 | | 0.734 (0.511 to 1.054) | 0.0943 |
| Male vs Female | 676 vs 253 | | 1.578 (1.214 to 2.053) | **0.0007** | | 579 vs 187 | | 1.494 (1.149 to 1.943) | **0.0028** |
| Smoking history yes vs no | 585 vs 286 | | 1.773 (1.374 to 2.288) | **<0.0001** | | 472 vs 201 | | 1.308 (1.022 to 1.674) | **0.0330** |
| Resection yes vs no | 363 vs 304 | | 0.591 (0.459 to 0.760) | **<0.0001** | | 45 vs 399 | | 0.312 (0.160 to 0.608) | **0.0006** |
| Adenocarcinoma vs others | 534 vs 394 | | 0.624 (0.503 to 0.775) | **<0.0001** | | 414 vs 350 | | 0.656 (0.529 to 0.814) | **0.0001** |
| Surgery as a part of initial therapy yes vs no | 348 vs 581 | | 0.611 (0.485 to 0.769) | **<0.0001** | | 53 vs 713 | | 0.419 (0.240 to 0.729) | **0.0021** |
| cCRT alone as initial therapy yes vs no | 247 vs 682 | | 0.972 (0.756 to 1.250) | 0.8247 | | 254 vs 512 | | 0.845 (0.671 to 1.063) | 0.1496 |
| cCRT alone as initial therapy vs sCRT alone as a part of initial therapy | 247 vs 82 | | 0.707 (0.475 to 1.052) | 0.0871 | | 254 vs 78 | | 0.729 (0.501 to 1.060) | 0.0983 |
| Trimodality as a part of initial therapy yes vs no | 120 vs 809 | | 0.638 (0.445 to 0.915) | **0.0145** | | 19 vs 747 | | 0.384 (0.143 to 1.029) | 0.0571 |
| **Multivariate Analyses** |  | |  |  | |  | |  |  |
| Age>65 vs ≤65 | 226 vs 311 | | 1.320 (0.993 to 1.755) | 0.0563 | | 187 vs 269 | | 1.275 (0.974 to 1.670) | 0.0770 |
| ECOG ≤1 vs 2/3/4 | 488 vs 49 | | 0.544 (0.358 to 0.826) | **0.0043** | | 406 vs 50 | | 0.623 (0.424 to 0.916) | **0.0162** |
| Male vs Female | 395 vs 142 | | 1.198 (0.745 to 1.927) | 0.4564 | | 347 vs 109 | | 1.126 (0.713 to 1.779) | 0.6112 |
| Smoking history yes vs no | 361 vs 176 | | 1.567 (1.007 to 2.437) | **0.0464** | | 323 vs 133 | | 1.061 (0.695 to 1.619) | 0.7845 |
| Adenocarcinoma vs others | 301 vs 236 | | 0.860 (0.632 to 1.171) | 0.3381 | | 250 vs 206 | | 0.775 (0.586 to 1.026) | 0.0750 |
| Surgery as a part of initial therapy yes vs no | 191 vs 346 | | 0.696 (0.477 to 1.014) | 0.0593 | | 26 vs 430 | | 0.419 (0.184 to 0.954) | **0.0382** |
| cCRT alone as initial therapy yes vs no | 156 vs 381 | | 0.600 (0.422 to 0.853) | **0.0045** | | 179 vs 277 | | 0.756 (0.572 to 0.999) | **0.0496** |
| Trimodality as a part of initial therapy yes vs no | 78 vs 459 | | 0.739 (0.434 to 1.260) | 0.2668 | | 7 vs 449 | | 1.231 (0.246 to 6.166) | 0.8005 |

AJCC=American Joint Committee on Cancer; cCRT=Concurrent chemoradiotherapy; CI=Confidence interval; ECOG=Eastern Cooperative Oncology Group; EGFRm=Epidermal Growth Factor Receptor mutation, EGFRwt=Epidermal Growth Factor Receptor wildtype mutation; HR=Hazard ratio; N=Number of patients^;^ NSCLC=Nonsmall cell lung cancer; sCRT=Sequential chemoradiotherapy; vs=Versus.

Values in bold indicate significant difference (p<0.05)
